# Mapping the global distribution of Buruli ulcer: a systematic review with evidence consensus

**DOI:** 10.1016/S2214-109X(19)30171-8

**Published:** 2019-07-01

**Authors:** Hope Simpson, Kebede Deribe, Earnest Njih Tabah, Adebayo Peters, Issaka Maman, Michael Frimpong, Edwin Ampadu, Richard Phillips, Paul Saunderson, Rachel L Pullan, Jorge Cano

**Affiliations:** Department of Disease Control, London School of Hygiene and Tropical Medicine, London, UK; Wellcome Trust Brighton and Sussex Centre for Global Health Research, Brighton and Sussex Medical School, Brighton, UK; National Yaws, Leishmaniasis, Leprosy and Buruli Ulcer Control Programme, Yaoundé, Cameroon; The National Tuberculosis, Leprosy and Buruli Ulcer Control Programme, Abuja, Nigeria; National Reference Laboratory for Buruli ulcer disease in Togo, Ecole Supérieure des Techniques Biologiques et Alimentaires (ESTBA), Laboratoire des Sciences Biologiques et des Substances Bioactives, Université de Lomé, Lomé, Togo; National Buruli Ulcer Control Program, Ghana Health Service, Accra, Ghana; School of Medical Sciences, Kwame Nkrumah University of Science and Technology, Kumasi, Ghana; National Buruli Ulcer Control Program, Ghana Health Service, Accra, Ghana; American Leprosy Missions, Greenville, SC, USA; Department of Disease Control, London School of Hygiene and Tropical Medicine, London, UK

## Abstract

**Background:**

Buruli ulcer can cause disfigurement and long-term loss of function. It is underdiagnosed and under-reported, and its current distribution is unclear. We aimed to synthesise and evaluate data on Buruli ulcer prevalence and distribution.

**Methods:**

We did a systematic review of Buruli ulcer prevalence and used an evidence consensus framework to describe and evaluate evidence for Buruli ulcer distribution worldwide. We searched PubMed and Web of Science databases from inception to Aug 6, 2018, for records of Buruli ulcer and *Mycobacterium ulcerans* detection, with no limits on study type, publication date, participant population, or location. English, French, and Spanish language publications were included. We included population-based surveys presenting Buruli ulcer prevalence estimates, or data that allowed prevalence to be estimated, in the systematic review. We extracted geographical data on the occurrence of Buruli ulcer cases and *M ulcerans* detection from studies of any type for the evidence consensus framework; articles that did not report original data were excluded. For the main analysis, we extracted prevalence estimates from included surveys and calculated 95% CIs using Byar’s method. We included occurrence records, reports to WHO and the Global Infectious Diseases and Epidemiology Network, and surveillance data from Buruli ulcer control programmes in the evidence consensus framework to grade the strength of evidence for Buruli ulcer endemicity. This study is registered with PROSPERO, number CRD42018116260.

**Findings:**

2763 titles met the search criteria. We extracted prevalence estimates from ten studies and occurrence data from 208 studies and five unpublished surveillance datasets. Prevalence estimates within study areas ranged from 3·2 (95% CI 3·1–3·3) cases per 10000 population in Côte d’Ivoire to 26·9 (23·5–30·7) cases per 10000 population in Benin. There was evidence of Buruli ulcer in 32 countries and consensus on presence in 12.

**Interpretation:**

The global distribution of Buruli ulcer is uncertain and potentially wider than currently recognised. Our findings represent the strongest available evidence on Buruli ulcer distribution so far and have many potential applications, from directing surveillance activities to informing burden estimates.

**Funding:**

AIM Initiative.

## Introduction

Buruli ulcer is a neglected tropical disease caused by the environmental pathogen *Mycobacterium ulcerans*. This disease primarily occurs in west and central Africa, but also in parts of Asia, South America, the western Pacific, and Australasia.^1,2^ It is considered an important public health problem because of the characteristic necrotic ulcers it causes, and the scarring and deformity that can persist after treatment.^3^ Although the mode of transmission of *M ulcerans* is not fully understood, contact with slow-flowing, stagnant, or disturbed water bodies is an important risk factor.^4^

Buruli ulcer was reported in 34 countries between 1960 and 2015,^4^ but there is no consensus on its current distribution. Ten countries reported a total of 1864 cases to WHO in 2016,^1^ but this number is recognised to reflect a small proportion of the total burden. Cross-sectional surveys in endemic countries have demonstrated under-reporting of Buruli ulcer,^5–7^ for reasons including the chronic, stigmatising nature of the disease, its rural distribution, patients’ poor access to health care or preference for traditional healers, and lack of awareness or resources within health systems.^4,8^ Misdiagnosis might also contribute to underdetection: Buruli ulcer has a range of non-specific presentations that can be confused with other skin conditions, especially in the absence of confirmatory tests.^9,10^ Therefore, available data do not provide a full or accurate representation of Buruli ulcer burden and distribution. These measures are essential for targeting of active case detection, which is a key part of control,3 and for directing resources for case management.

Estimation of the global burden and population at risk of Buruli ulcer requires detailed information on the geographical limits and prevalence of the disease. We aimed to synthesise available data on prevalence and occurrence of Buruli ulcer and environmental occurrence of *M ulcerans*, and to systematically review population-based studies reporting the prevalence of Buruli ulcer to provide a descriptive analysis of Buruli ulcer epidemiology in known endemic areas. We aimed to use an evidence consensus approach^11,12^ to delineate the overall distribution of previously reported cases and to quantify the strength of evidence for Buruli ulcer presence or absence in every country worldwide.

## Methods

### Search strategy and selection criteria

We did a systematic review of Buruli ulcer prevalence and used an evidence consensus framework to describe and evaluate evidence for Buruli ulcer distribution worldwide. Data sources included peer-reviewed scientific literature; conference proceedings, conference abstracts, and government reports (grey literature); data reported to WHO between 2007 and 2016;^1^ data reported through the Global Infectious Diseases and Epidemiology Network (GIDEON);^13^ and surveillance datasets from national Buruli ulcer programmes in Cameroon, Ghana, Nigeria, and Togo. Peer-reviewed literature was identified from searches of PubMed and Web of Science databases from inception to Aug 6, 2018. Additional publications were identified from reference lists of identified papers.

We used the search terms “Buruli ulcer*” OR (“Mycob* AND ulcer*”) OR “Bairnsdale ulcer”. There were no limits on publication date, participant population, study type, or location (details in appendix). English, French, and Spanish language publications were included. Population-based Buruli ulcer surveys were included in the systematic review if they reported the prevalence of Buruli ulcer within a defined geographical area or information that allowed prevalence to be calculated. Publications were eligible for inclusion in the evidence consensus if they reported geographical locations with evidence of *M ulcerans* infection in humans or animals, or detection of *M ulcerans* in animal and environmental samples. Articles that did not report original data were excluded.

One author (HS) screened titles to exclude non-relevant publications and screened abstracts of selected records to identify papers that apparently fulfilled selection criteria. We read full texts of selected articles to identify studies meeting the selection criteria. Studies that recruited patients from health facilities or used strains of *M ulcerans* isolated from clinical samples were included in the evidence consensus framework only if patients’ home addresses were provided. Data from people with Buruli ulcer who had recorded travel history to several endemic regions were excluded. If a dataset was duplicated in numerous papers, the most comprehensive version was included.

### Data extraction

Data from surveillance datasets and selected publications were extracted into a bespoke Microsoft Excel spreadsheet used for the Global Atlas of Helminth Infections.^14^ The original spreadsheet was piloted on a subset of studies and then developed. Authors were contacted for additional data if community-level results were not presented. Data extraction was done by a single author (HS) and checked by a second one (JC). Data extracted included the number or prevalence of cases; the sample size and survey coverage (for population-based studies); the case detection method (survey, case search, or passive detection); the recording date; the diagnostic procedure, including any confirmatory tests (PCR for *M ulcerans* gene targets, Ziehl-Neelsen staining, culture for *M ulcerans*, and histopathological analysis), and their results; and the location of origin (patient residence or endemic area visited if the case originated from a non-endemic area). Areas described as endemic, with no information on case detection, were not included.

Data extracted on environmental detection of *M ulcerans* included sample date and location; sample type (water, soil, plant, or animal [clinical or faecal]); taxonomic details for animal samples; confirmatory tests; and number of samples tested and number positive.

Geographical coordinates of occurrence locations were extracted if they were provided in the publication. Otherwise, point locations were georeferenced remotely (appendix). Point locations that could not be georeferenced were linked to the lowest administrative level provided in the publication. Polygon areas corresponding to first and second administrative divisions were linked to units defined in the Database of Global Administrative Areas.

### Summary measures

The main summary measure for the systematic review was Buruli ulcer prevalence. The quality of prevalence studies was assessed with a framework based on the Newcastle-Ottawa scale,^15^ adapted from a systematic review of podoconiosis prevalence^16^ (appendix). This framework took account of the sampling frame, survey coverage, diagnostic specificity, and statistical analysis. The risk of outcome bias was assessed according to whether sampling was done at random or using convenience sampling within the study area. The number of studies from each country, relative to the number of cases reported to WHO, was used as an indicator of geographical bias between studies.

The main outcome measures for the evidence consensus framework were Buruli ulcer and *M ulcerans* occurrence. Occurrence locations were assigned local-level and national-level quality scores reflecting contemporariness and specificity (appendix). We used the number of studies included in the evidence consensus framework, and the number of studies reporting laboratory confirmation, as indicators of geographical bias in reporting and study quality.

### Data analysis

**Figure 1:**
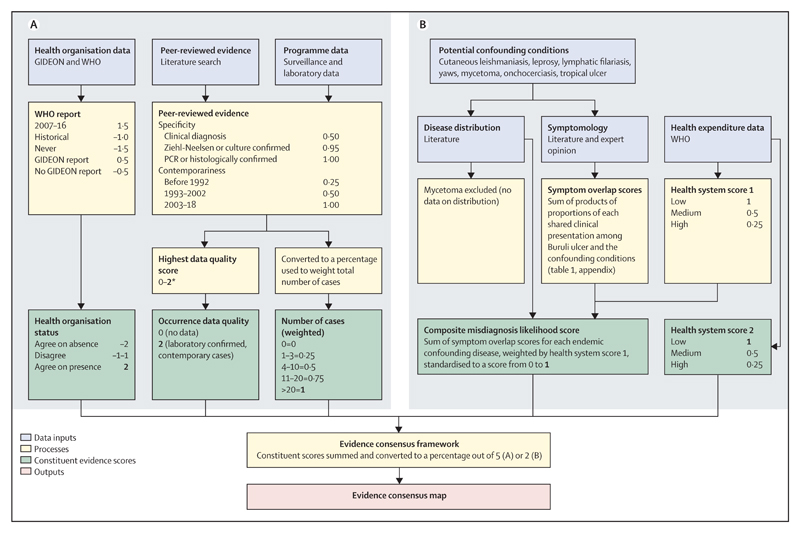
Evidence consensus framework used to assess strength of evidence for Buruli ulcer presence and absence at national level (A) Framework for all countries. (B) Framework for countries with no evidence of reported cases. Numbers in bold show each constituent’s maximum score. GIDEON=Global Infectious Diseases and Epidemiology Network. *Score was adjusted post-hoc for countries from which *Mycobacterium ulcerans* strains had been isolated, if no cases meeting inclusion criteria were identified.

**Figure 2:**
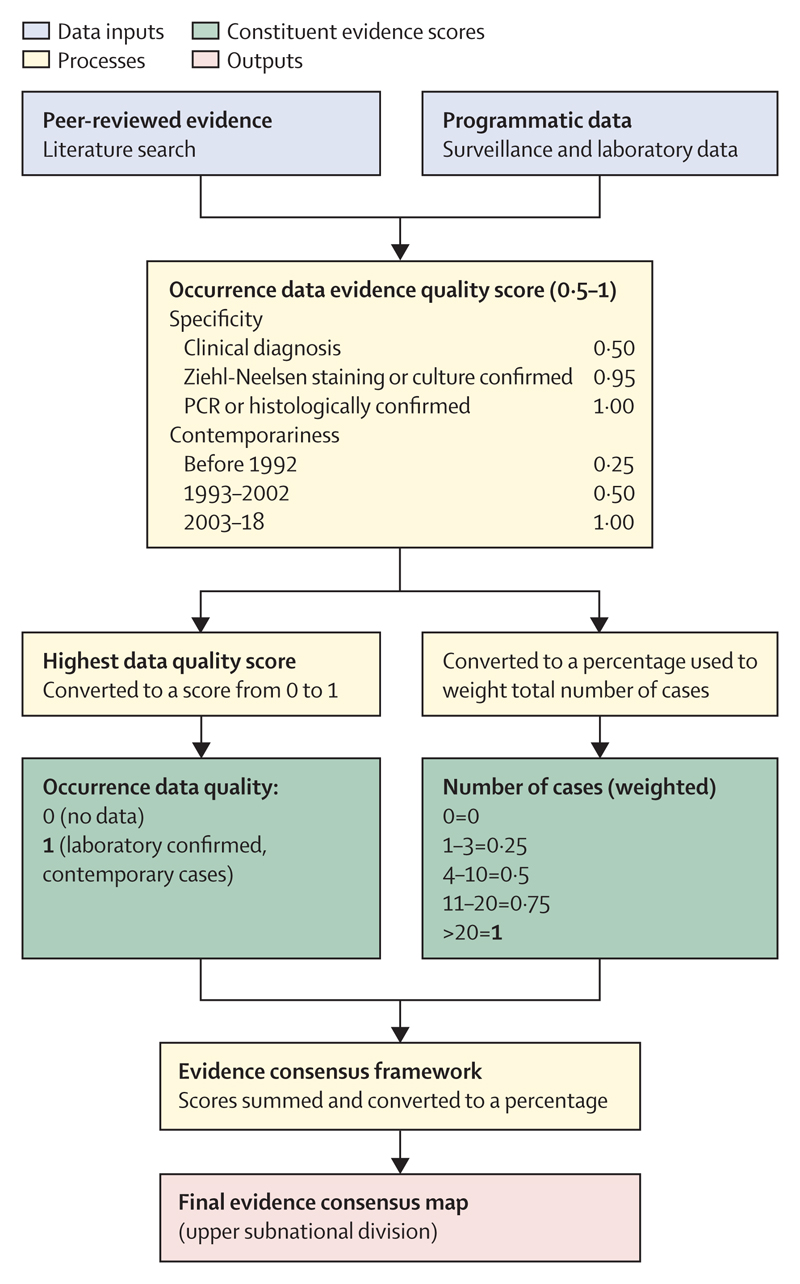
Evidence consensus framework used to assess strength of evidence for Buruli ulcer presence at subnational level Numbers in bold show each constituent’s maximum score.

We extracted prevalence estimates from included surveys and calculated 95% CIs using Byar’s method.^17^ We synthesised occurrence data through an evidence consensus approach using a weighted scoring system, following that used to determine the global distribution of other diseases.^11,12^ Separate frameworks were used to assess the evidence for Buruli ulcer presence or absence at the national level (figure 1), evidence for Buruli ulcer presence at the subnational level (figure 2), and evidence for environmental occurrence of *M ulcerans* at the subnational level (appendix).

The major features for the national evidence framework were health reporting organisations (countries were assigned a score based on recent and historical reporting to WHO and reports through GIDEON); occurrence data quality (each country was assigned the highest data quality score of occurrence records within it); number of cases (the number of cases reported at each location was weighted by the local-level data quality score, and the weighted totals were aggregated to national level); and evidence for absence. In countries with no cases reported, the consensus score was designed to quantify the evidence for Buruli ulcer absence, reflecting the possibility of under-reporting due to weak surveillance capacity or misdiagnosis as known endemic diseases with similar presentations^18^ (confounding diseases; figure 1B). As a proxy for surveillance and diagnostic capacity, health expenditure reported by WHO^19^ was categorised as low (<US$100), medium ($100–$499), or high (≥$500), following the approach of previous authors and supported by evidence that higher health expenditure is associated with better health system performance.^20^

The confounding diseases with available evidence on their global distribution were cutaneous leishmaniasis,^12,21^ leprosy,^22^ lymphatic filariasis,^14^ onchocerciasis,^23^ tropical ulcer,^2^ and yaws.^24^ Estimates of the frequencies of the common presentations of these diseases and Buruli ulcer were obtained from literature review and expert opinion (Saunderson P, unpublished).^23,25–27^ For each confounding disease, the frequency of each presentation shared with Buruli ulcer was multiplied by the frequency of the presentation among Buruli ulcer cases, and the products were summed to generate a symptom overlap score (appendix). For each country, the symptom overlap scores for its endemic confounding diseases were summed, then downweighted if health expenditure was high or medium. This score was added to an ordinal health expenditure score reflecting likelihood of underdetection or non-reporting.

For the subnational level, each upper administrative level was assigned the highest local-level evidence quality score of the occurrence records that fell within it, or within 5 km distance of its boundaries, and a score reflecting total number of cases within the unit (figure 2). Environmental detection records for *M ulcerans* were assigned to the upper administrative unit that they fell within. Each unit was assigned the highest evidence quality score of records within it, and a score reflecting the total number of detection records within it, weighted by evidence quality score (appendix).

This study is registered with PROSPERO, number CRD42018116260.

### Role of the funding source

The AIM Initiative facilitated connections with disease control programmes for data transfer but neither it nor the Wellcome Trust had any role in study design, data collection, data analysis, data interpretation, or writing of the report. The corresponding author had full access to all the data in the study and had final responsibility for the decision to submit for publication.

## Results

**Figure 3:**
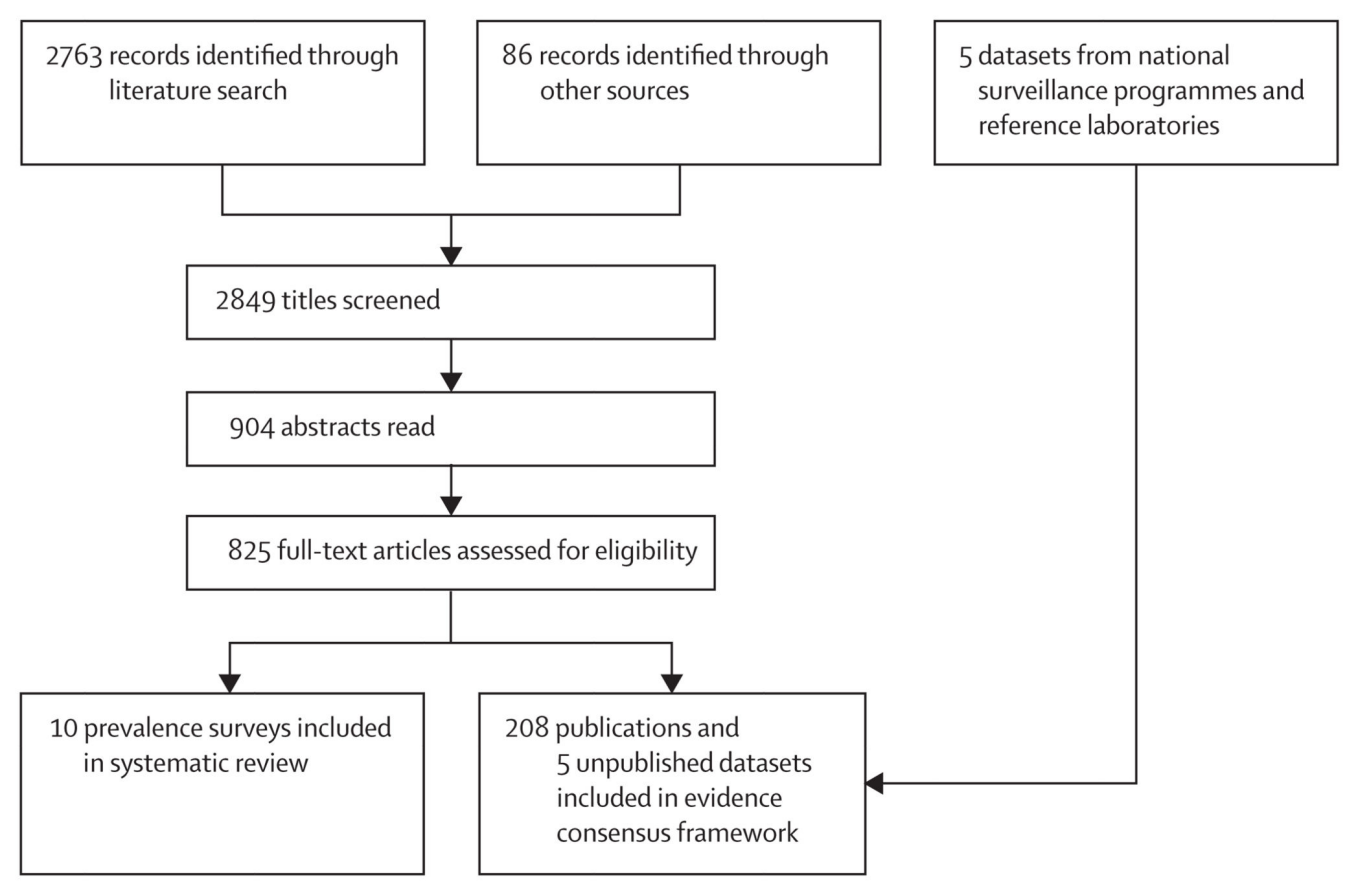
Selection of eligible studies

The literature search identified 2763 records after deduplication (figure 3). Another 86 records were identified through other sources. The most common reason for exclusion was scarcity of information on patient origin. Full text was unavailable for 46 studies. Ten Buruli ulcer prevalence surveys were included in the systematic review.^7,8,28–33^ Occurrence data were extracted from 208 publications (of which 190 included data on human cases and 34 included data on *M ulcerans* in environmental or animal samples) and five unpublished surveillance datasets.

Three surveys done in Cameroon, two in each of Benin, Côte d’Ivoire, and Ghana, and one in the Democratic Republic of the Congo were included (table 1). The largest survey was done in Côte d’Ivoire, covering an estimated 14 500 000 people.^5^ Seven surveys provided explicit details on the sampling frame. All surveys were community based and aimed to reach the entire population of chosen communities. Seven surveys covered the entire study area, one surveyed randomly selected communities within the study area, one surveyed a convenience sample of communities, and one used random and convenience sampling. Only one reported the survey coverage.^8^ Five reported laboratory confirmation of all or a subset of cases, and five used clinical case definitions. Only one study reported prevalence with 95% CIs.^8^

Overall prevalence estimates within the study area ranged from 3·2 (95% CI 3·1–3·3) cases per 10 000 population in Côte d’Ivoire to 26·9 (23·5–30·7) cases per 10 000 in Benin (table 1). The highest reported community prevalence of Buruli ulcer was 2200 cases per 10 000 population, recorded in a village in Amansie West district in Ghana.^32^

Human cases were recorded from 32 countries and inferred for two further countries (Iran and Malaysia) from which strains were reported to have been isolated.^37,38^ 33 794 (94·9%) of 35 595 cases were from the African (AFRO) region, 1740 (4·9%) cases were from the Western Pacific (WPRO) region, 60 (0·2%) were from the American (AMRO) region, and one (<0·1%) was from the Eastern Mediterranean (EMRO) region. Evidence of *M ulcerans* in environmental and animal samples was reported from nine countries. A summary of data extracted from all publications is provided in the appendix. Cases were recorded from 1952 to 2017, with the greatest number detected in 1999 (3401). From 1952 to 1998, between zero and five countries each year had evidence of Buruli ulcer based on peer-reviewed literature. The disease was identified in nine countries in 1999. Including data reported to WHO, from 2007 to 2016, between 12 and 18 countries each year had evidence of Buruli ulcer.

Laboratory confirmation of at least one case was reported by 134 (70·5%) of 190 selected studies including data on human cases, and 116 (61·1%) used PCR. However, most occurrence records (3165 [53·0%] of 5970) were categorised as clinically diagnosed only, because laboratory results were not disaggregated by unique locations.

Symptom overlap scores for the confounding diseases are shown in table 2. Tropical ulcer had the highest score, reflecting the high frequency of ulcers among Buruli ulcer and tropical ulcer.^2,33^ Buruli ulcer was considered less likely to be misdiagnosed as cutaneous leishmaniasis or yaws, which present a lower frequency of ulcerous forms.^25,26^ Onchocerciasis, leprosy, and lymphatic filariasis had symptom overlap scores of less than 6%.

**Figure 4:**
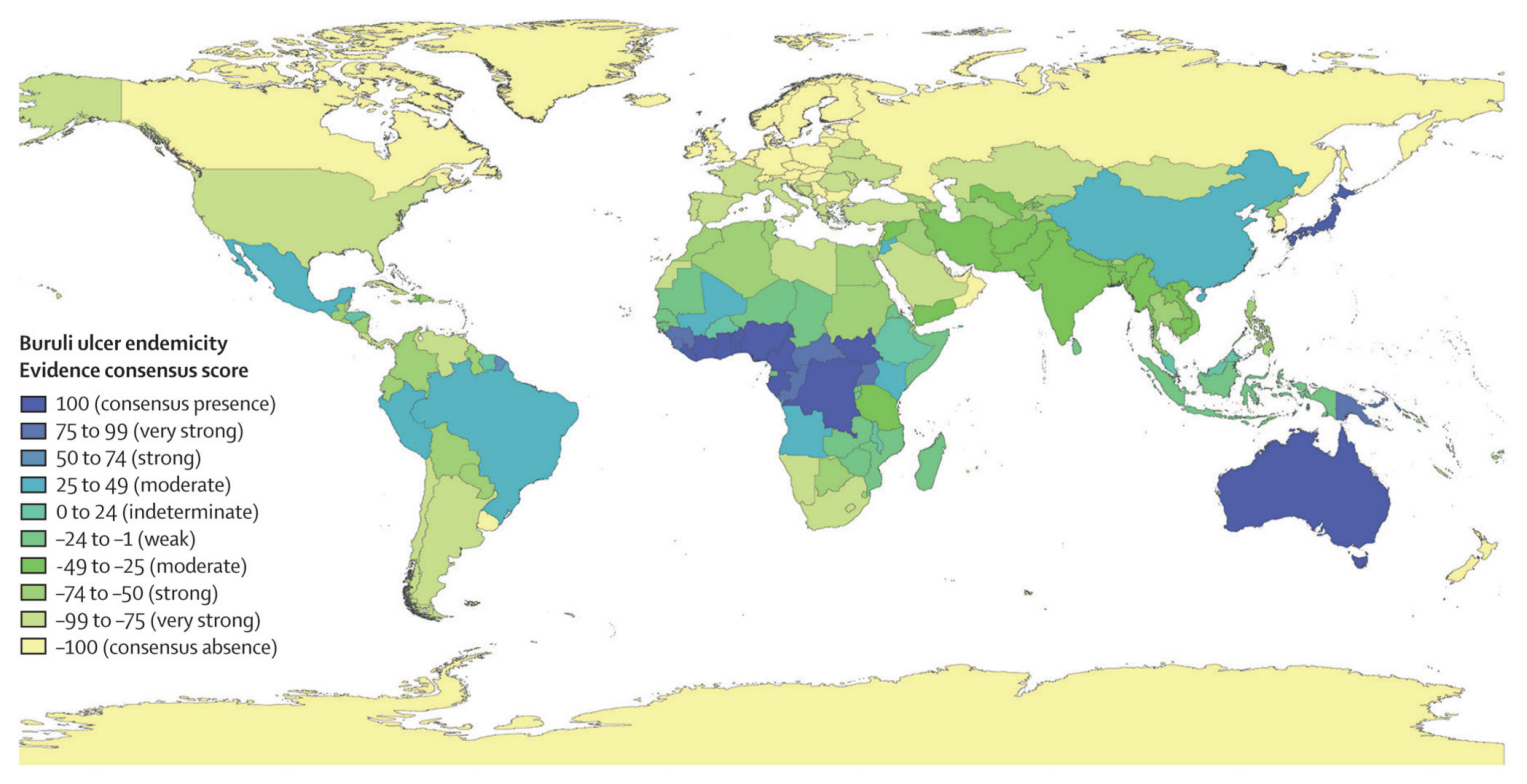
Evidence consensus for Buruli ulcer presence and absence worldwide

Full results of the evidence consensus framework are provided at country level in the appendix. We identified consensus on Buruli ulcer presence in 12 countries, which collectively reported 34 890 cases to WHO from 2007 to 2016 (96·5% of all 36 164 cases reported to WHO in this period). Six countries reported cases to WHO from 2007 to 2016, but did not reach consensus of evidence for Buruli ulcer endemicity because of scarcity of information on case confirmation. Australia and Japan were the only non-African countries with consensus on presence (figure 4).

The African countries with evidence of Buruli ulcer were mostly clustered in a block covering much of central and west Africa. Countries around this block generally had weaker evidence for absence, with a higher number of endemic confounding diseases and lower health expenditure than did countries further from endemic areas. In the AMRO region, evidence of Buruli ulcer was strong in French Guiana and Peru, and moderate in Brazil, Mexico, and Suriname. Despite strong evidence of Buruli ulcer cases from French Guiana in literature reports, the disease has never been reported to WHO, so full consensus on endemicity was not reached through the framework. There was moderate evidence for Buruli ulcer in China. Endemicity status was indeterminate in Burkina Faso, Ethiopia, Honduras, Indonesia, Malawi, Malaysia, and Suriname. Niger, Eritrea, The Gambia, and Mauritania, all in the AFRO region, had the weakest evidence for absence, being endemic for cutaneous leishmaniasis and tropical ulcer, and having low healthcare expenditure.

**Figure 5:**
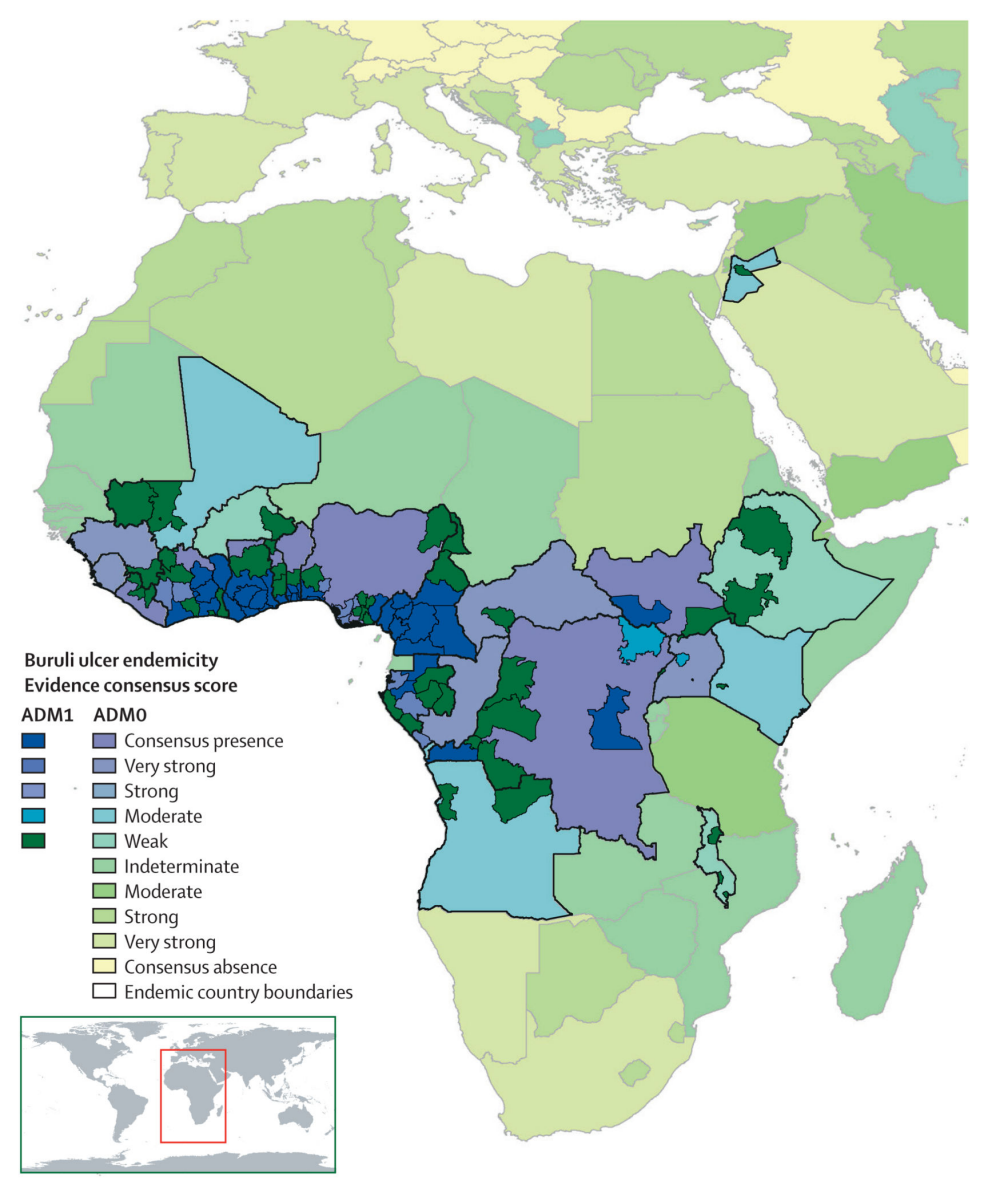
Evidence for Buruli ulcer endemicity at national and upper subnational levels in Africa ADM0=national administrative division. ADM1=upper subnational administrative division.

**Figure 6:**
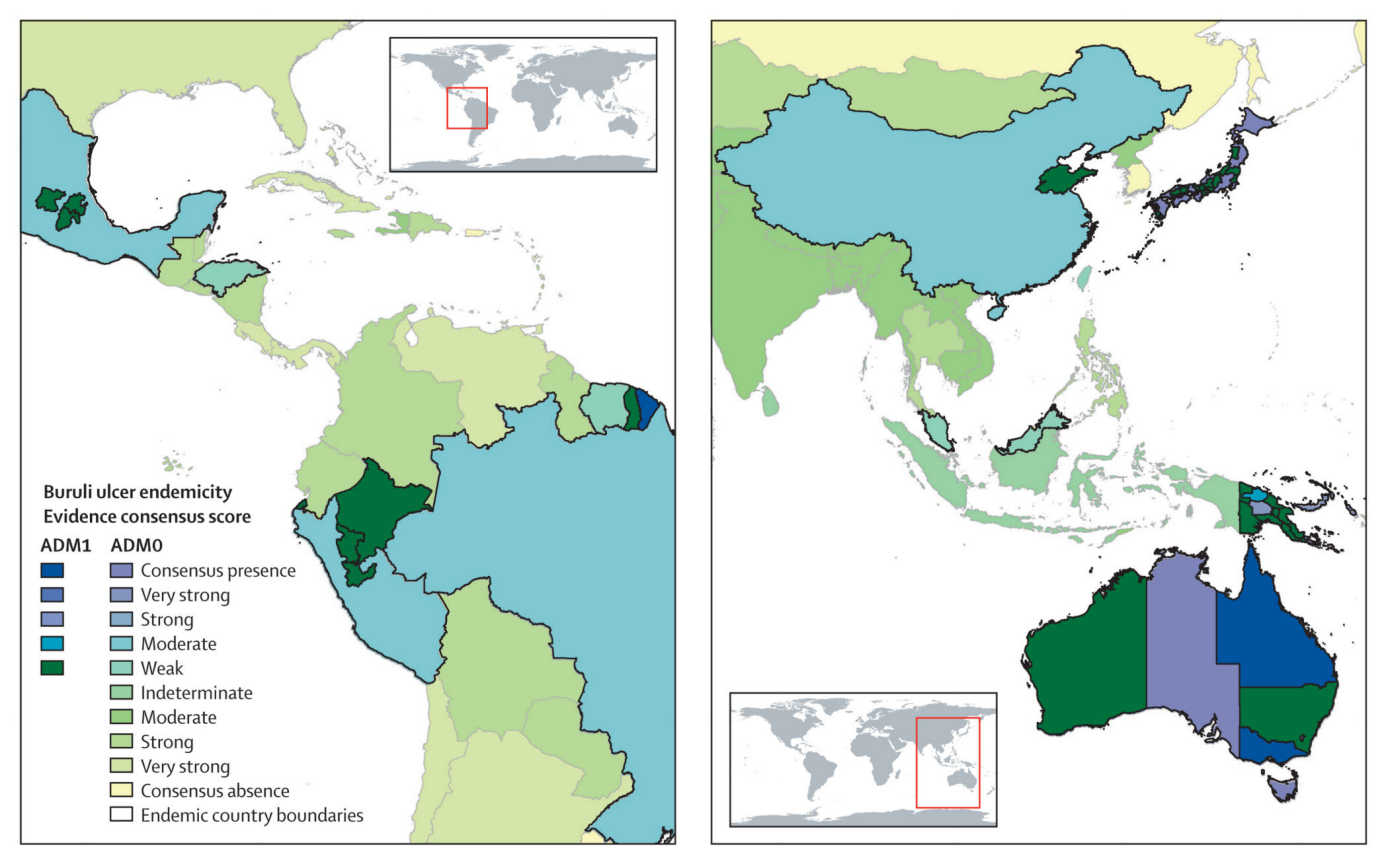
Evidence for Buruli ulcer endemicity at national and upper subnational levels in Central and South America and the Pacific Region ADM0=national administrative division. ADM1=upper subnational administrative division.

Subnational areas with evidence for endemicity were mostly clustered within equatorial, humid tropical, and tropical climate zones of west and central Africa (figure 5). Areas with evidence for Buruli ulcer in eastern, southern, and non-coastal central Africa, and other parts of the world, were more isolated (figures 5, 6).

**Figure 7:**
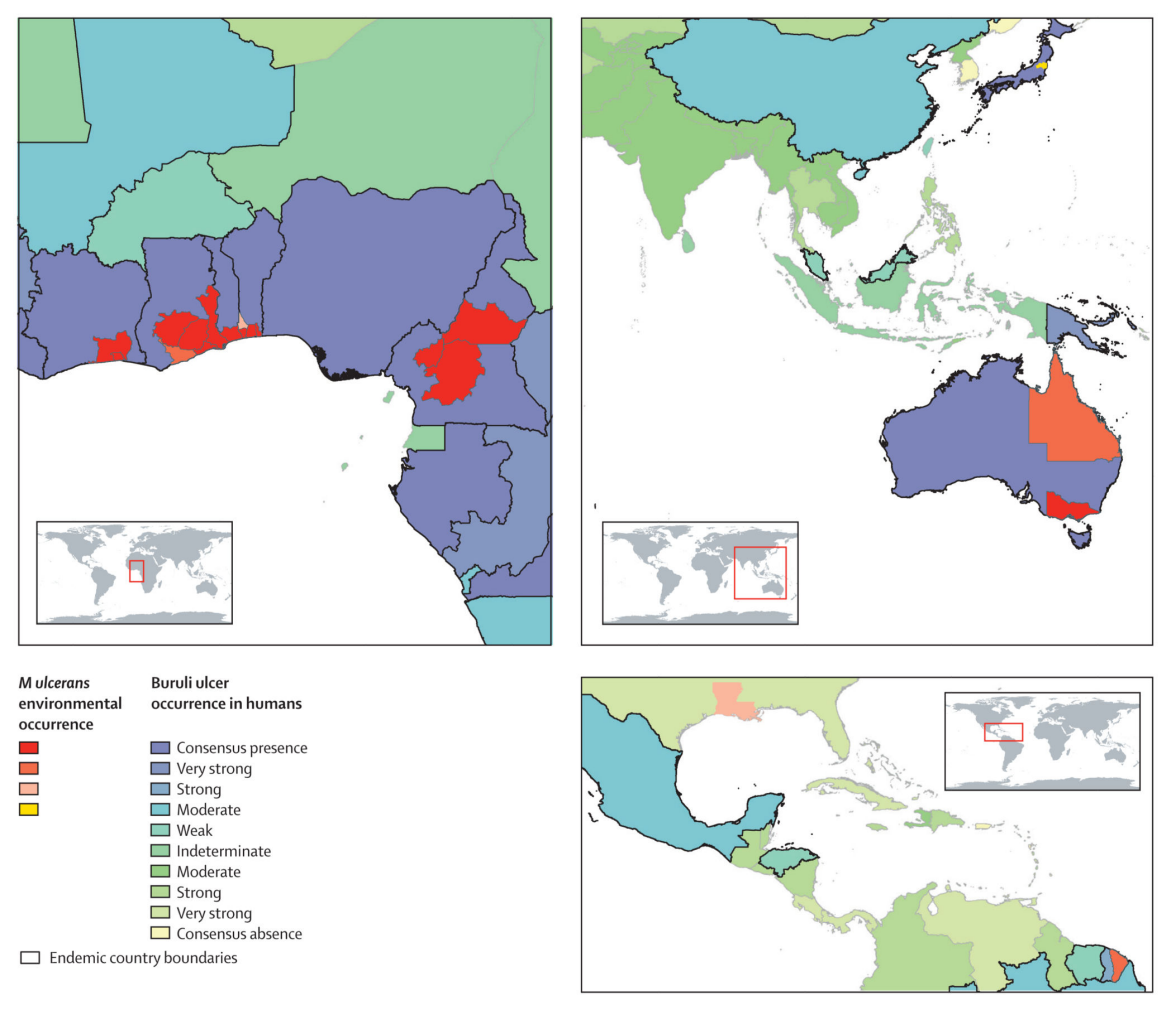
Evidence for environmental occurrence of *Mycobacterium ulcerans* at upper subnational level and for Buruli ulcer endemicity at national level in west and central Africa, the western Pacific region, and South America

The areas with evidence of *M ulcerans* in animal and environmental samples are shown in figure 7. Buruli ulcer disease was reported in wild and domestic animals in Australia, Benin, Cameroon, and Ghana, and *M ulcerans* DNA has been detected in faecal samples from animals in Australia (details and references in appendix). DNA from mycolactone-producing environmental bacteria has been identified in biotic and abiotic samples from bodies of water in eight countries endemic for Buruli ulcer and in the USA (details and references in the appendix). However, whether the American strains would be capable of causing Buruli ulcer disease in humans is unclear.

## Discussion

We have collated available data on Buruli ulcer prevalence and occurrence, and evidence of *M ulcerans* in animals and the environment. The evidence consensus framework applied has allowed us to expand on existing maps of Buruli ulcer distribution^2,39^ in several ways. The maps presented include evidence from a wider range of sources, provide finer resolution, and quantify the strength of evidence for Buruli ulcer presence, as well as the strength of evidence of absence where Buruli ulcer has not been reported.

There have been few Buruli ulcer prevalence surveys, and most of those identified did not report detailed statistical analysis or indicators such as coverage. We did not undertake a meta-analysis because of the heterogeneous nature of compiled studies. Furthermore, most studies included were done in areas assumed to have a high local prevalence of Buruli ulcer, so a summary prevalence would probably overestimate the disease burden in the overall population.

Prevalence estimates reported by population-based studies were high relative to incidence data reported to WHO. This difference is likely to reflect under-reporting of Buruli ulcer through routine systems, but the population-based studies included might have overestimated Buruli ulcer prevalence as a result of sampling bias. Two of the ten studies included^7,33^ used convenience sampling as part of the study design, which implies a risk of bias in the estimated prevalence. Five studies reported clinical diagnosis according to WHO guidelines and five used laboratory testing to confirm all or a subset of cases. There was geographical bias across the studies included, representing only five countries of the 32 identified as having evidence for Buruli ulcer.

Our investigation identified consensus on Buruli ulcer presence in 12 of 18 countries that reported Buruli ulcer cases to WHO from 2007 to 2016. However, the maps presented demonstrate remaining uncertainty on the global distribution of Buruli ulcer. There was indeterminate or moderate-quality evidence of Buruli ulcer in 15 countries that had not reported data to WHO from 2007 to 2016.

The national and subnational evidence consensus maps demonstrate large contiguous areas of potential endemicity, both within and between countries, particularly in central and west Africa. Evidence for Buruli ulcer presence was generally strongest in these contiguous areas, which is likely to be partly due to environmental similarity in terms of suitability and partly due to increased emphasis on case detection in areas established as endemic.

The area of Buruli ulcer presence defined by the subnational map of Buruli ulcer distribution in Africa (figure 5) was more restricted than that defined by the map of national-level endemicity (figure 4). This finding reflects the focal and restricted distribution of Buruli ulcer,^40^ and the lower availability of data at the subnational level: in some countries, the only available data were those reported to WHO, with no information on subnational distribution. Given the recognised scale of Buruli ulcer under-reporting, it is likely that this map underestimates the scale of Buruli ulcer distribution.

Countries that had not reported Buruli ulcer cases, but were close to those that had, generally had weaker evidence for absence than countries located further from areas of Buruli ulcer endemicity. This trend was apparent in Africa, South America, and the southeast Asia and western Pacific regions, and reflects spatial clustering of countries with lower health expenditure and numerous co-endemic tropical diseases, irrespective of their evidence for Buruli ulcer. The proximity of Buruli ulcerendemic countries to those with lowest evidence for Buruli ulcer absence adds further weight to the possibility that Buruli ulcer might occur undetected in the latter group, as a result of cross-border transmission and environmental similarity of neighbouring countries.

Although the maps provide finer detail on the distribution of Buruli ulcer than do current official maps, they still mask the underlying epidemiology of Buruli ulcer. Areas identified as endemic might in fact contain only a few localised cases of Buruli ulcer and be mostly unsuitable for the disease. Because of the focal nature of Buruli ulcer,^40^ point-level data on disease occurrence are needed to support investigation into its spatial epidemiology. It is hoped that the maps and assembled geographical dataset will support such research in the future.

Studies on environmental occurrence of *M ulcerans* were limited in number, and many did not apply sufficiently specific tests to differentiate *M ulcerans* from other environmental mycobacteria. Therefore, the maps of evidence for environmental occurrence of *M ulcerans* do not provide a complete representation of environmental suitability for the bacterium. Although we assigned the maximum possible evidence quality score to clinical cases confirmed by PCR and environmental occurrences confirmed by quantitative PCR, these tests still entail a risk of false positives, as demonstrated by an external quality assessment including several reference laboratories that performed confirmatory testing in studies we included.^41^

There was substantial geographical bias in the occurrence records, reflecting different levels of research and surveillance activity between countries. Further analysis of the data underlying this work should account for this bias. In the context of this study, this bias is expected to have affected areas where there were few studies, but not areas where there were many studies, since additional studies would not change the outcome measure unless they provided higher-quality data.

The areas with highest consensus for presence are presumably most suitable for Buruli ulcer transmission and would be targets for surveillance and research since they represent known disease foci. Some countries with strong evidence for Buruli ulcer are not shown in the current WHO map of Buruli ulcer,^39^ demonstrating that the disease is likely to be more widely distributed than the official map suggests. This finding has important implications for understanding and communicating the global burden of Buruli ulcer. We have also expanded on the WHO map of Buruli ulcer distribution by qualitatively grading the strength of evidence for endemicity. In doing so, we have identified numerous countries with moderate or indeterminate evidence of Buruli ulcer, and those with weakest evidence for its absence, which might require further investigation to clarify the global distribution of Buruli ulcer. Active case finding in areas that have previously reported Buruli ulcer, and close to those currently reporting, should be prioritised. The assembled point-level dataset represents a novel resource for continent-wide exploration of environmental and biological predictors of Buruli ulcer, and estimation of the global burden and population at risk. The information provided by investigations such as these will help to target future control efforts and evaluate their impact.

## Supplementary Material

Supplementary appendix 1

## Figures and Tables

**Table 1:** Characteristics of population-based Buruli ulcer prevalence surveys included in the systematic review

	Country	Year of survey	Location	Study design	Case ascertainment	Active cases	Sample size	Prevalence per 10 000 population (95% CI)	Quality score
Johnson et al (2005)^34^	Benin	2004	Lalo commune	Exhaustive preparatory phase followed by validation of suspected cases	Clinical diagnosis following WHO guidelines	160	86 819	18·4 (15·7–21·5)	4
Sopoh et al (2010)^29^	Benin	2006	Zè district	Exhaustive preparatory phase followed by validation of suspected cases	Clinical diagnosis following WHO guidelines	222	82 450	26·9 (23·5–30·7)	4
Noeske et al (2004)^7^	Cameroon	2001	Ayos and Akonolinga health districts	Exhaustive survey in convenience sample of communities with suspect cases	Clinical diagnosis, a subset confirmed by PCR or Ziehl-Neelsen staining	202	98 500	20·5 (17·8–23·5)	2
Porten et al (2009)^8^	Cameroon	2007	Akonolinga district	Exhaustive survey in a random selection of communities	Clinical diagnosis following WHO guidelines, active and total cases reported separately	56	26 679	21·0 (15·9–27·3)	5
Bratschi et al (2013)^35^	Cameroon	2010	Bankim Health District	Exhaustive survey of health district	Clinical diagnosis, a subset confirmed by PCR	25	48 962	5·1 (3·3–7·5)	3
Kanga (2001)^36^	Côte d’Ivoire	1995	Côte d’Ivoire	Exhaustive survey of entire country	Suspect cases identified by community health workers, confirmed by clinicians	4642	14 500 000	3·2 (3·1–3·3)	2
Ecra et al (2005)^30^	Côte d’Ivoire	1998	Zoukoougbeu subprefecture	Exhaustive survey of entire subprefecture	Nodules detected clinically, Mycobacterium ulcerans confirmed by histopathological analysis	54	47 742	11·3* (8·5–14·8)	3
Mavinga Phanzu et al (2013)^31^	Democratic Republic of the Congo	2008	Kimpese and Nsona-Mpangu Rural Health Zones	Exhaustive preparatory phase followed by validation of suspected cases	Clinical diagnosis following WHO guidelines, a subset confirmed by PCR	259	237 418	10·9 (9·6–12·3)	6
Amofah et al (1993)^32^	Ghana	1991	Amansie West district	Exhaustive survey of entire district	Clinical diagnosis, a subset confirmed by Ziehl-Neelsen staining	90	130 000	6·9 (5·6–8·5)	4
Ampah et al (2016)^33^	Ghana	2013	Ofin River valley	Exhaustive survey in random sample (n=10) and convenience sample (n=3) of communities within 5 km of the Ofin River	Clinical diagnosis in following WHO guidelines, a subset confirmed by PCR	7	20 390	3·4 (1·4–7·1)	6

* Prevalence of nodules only, did not include other forms of Buruli ulcer.

**Table 2:** Symptom overlap scores (0–100) for diseases whose symptoms can also be caused by Buruli ulcer

	Summed score
Tropical ulcer	70·9
Cutaneous leishmaniasis	35·0
Yaws	16·3
Onchocerciasis	5·7
Leprosy	3·6
Lymphatic filariasis	0·5

## Research in context

### Evidence before this study

We searched PubMed and Web of Science databases from inception to Aug 6, 2018, using the search terms “Buruli ulcer*” OR (“Mycob* AND ulcer*”) OR “Bairnsdale ulcer”. English, French, and Spanish language publications were included. We identified two systematic reviews on Buruli ulcer, neither of which was spatially focused. There were 13 non-systematic reviews, two of which included a literature search to collate evidence on the global distribution of Buruli ulcer infection, and presented the results in a map and a narrative summary, respectively. Five reviews used WHO-reported data to show the global distribution of Buruli ulcer. The Global Infectious Diseases and Epidemiology Network has mapped the Buruli ulcer distribution reports, which provides a broader evidence base, but the evidence in many countries is weak. Our understanding of global Buruli ulcer distribution is incomplete: poor access to health care and diagnostics, overburdened health systems, and weak surveillance systems and reporting capacity contribute to underdetection and under-reporting of Buruli ulcer.

### Added value of this study

To our knowledge, this is the first systematic review of Buruli ulcer prevalence and distribution worldwide. We compiled data from a wide range of sources, including the peer-reviewed and grey literature, WHO reports, and previously unpublished surveillance datasets. We used a systematic framework to grade the strength of evidence for Buruli ulcer presence, based on consensus between all data sources. This approach accounted for the specificity of diagnostic case definitions and reporting dates. We found evidence of Buruli ulcer occurrence in 32 countries, of which 18 had reported cases to WHO between 2007 and 2016. We identified consensus on Buruli ulcer presence in 12 countries, which reported a total of 34 890 cases to WHO from 2007 to 2016. Given the scale of under-reporting, absence of data on Buruli ulcer cannot be assumed to reflect disease absence. We have therefore expanded on previous work by grading evidence for absence of Buruli ulcer in countries that have not previously reported the disease. Countries with weak health systems and surveillance capacity might be failing to detect Buruli ulcer cases, or misdiagnosing them as other conditions. We calculated scores to reflect these possibilities using health expenditure values as a proxy for surveillance and diagnostic capacity, and accounting for the co-endemicity of diseases sharing clinical features with Buruli ulcer.

### Implications of all the available evidence

Our current understanding of Buruli ulcer distribution is incomplete: many countries that have reported data to WHO in the past decade lack published evidence of confirmed cases, whereas other countries with demonstrated evidence of Buruli ulcer transmission have not reported data to WHO. Countries with evidence of Buruli ulcer are mostly clustered in Africa. Many of these countries border countries with no evidence of cases, but with weak health systems and multiple co-endemic skin diseases, potentially masking incident Buruli ulcer cases. Further analysis, including ecological modelling, might help to further elucidate the full distribution of Buruli ulcer. Intensified active case finding should be prioritised in areas with weaker evidence, to better inform delivery of targeted interventions.
