# Supplementary material for: Mapping the global distribution of Buruli ulcer: a systematic review with evidence consensus
Source: Lancet Glob Health. Author manuscript; Available in PMC 2019 Jul 8. (PMC6614043; doi:10.1016/S2214-109X(19)30171-8)
Supplement: Supplementary appendix 1 [file EMS83482-supplement-Supplementary_appendix_1.pdf]

THE LANCET
Global Health

Supplementary appendix

This appendix formed part of the original submission and has been peer reviewed. We post it as supplied by the authors.

Supplement to: Simpson H, Deribe K, Tabah EN, et al. Mapping the global distribution of Buruli ulcer: a systematic review with evidence consensus. *Lancet Glob Health* 2019; **7:** e912–22.

Supplementary Methods

S.1. Protocol for Assembly of the Buruli Ulcer Database

Overview

The BU database was compiled through a systematic search of peer-reviewed literature and inclusion of routine surveillance data collected by control programmes responsible for BU in endemic countries. The database includes occurrence records of BU disease in humans and animals, and of evidence of Mycobacterium ulcerans detection in environmental and animal samples, each linked to a point or polygon location.

S.1.1. Peer-reviewed Literature Search

PubMed was searched using the terms:
(Buruli ulcer[Title/Abstract]) OR (Mycob*[Title/Abstract] AND ulcer*[Title/Abstract]) OR (Bairnsdale ulcer[Title/Abstract])
The Web of Science was searched using the terms:
TS=Buruli ulcer* OR TS=(Mycob* AND ulcer*) OR TS= (Bairnsdale ulcer)
The searches were updated in August 2018. There were no limits on study dates. Data were included from English, French, and Spanish language publications.
Reference lists of selected publications were screened.
Reports of relevant conferences and meetings were reviewed for relevant data.

The main outcome of interest was evidence of occurrence of Buruli ulcer or *M. ulcerans* infection in humans in any country, with no limit on detection date.
Suspect, clinical, and laboratory-confirmed cases of BU were all included, as were cases of serological evidence of *M. ulcerans* infection in the absence of clinical signs.
Imported cases were included if their place of infection was given. Imported cases with travel history to multiple BU-endemic regions were excluded.
Data extracted from publications presenting evidence of human infection with *M. ulcerans* included: i) the number or prevalence of cases identified (the minimum if a range was given), ii) the method of case ascertainment (e.g. survey, case search, passive detection), iii) the recording date (the maximum if a range was given), iv) the diagnostic procedure, including any confirmatory tests applied (PCR *M. ulcerans* gene targets; Ziehl Neelsen (ZN) staining; culture for *M. ulcerans*; histopathological analysis) and their results, and v) the location of origin (patient residence or endemic area visited if the case originated from a non-endemic area). Geographical information extracted from publications included geographical coordinates, site name, and description. If multiple cases were reported from a single geographical location, the cases were aggregated to a single occurrence location and by year.

If an area was described as ‘endemic’ but case numbers were not stated, the minimum number of cases was recorded as 1.
We also included occurrences of BU disease in animals, and detection of *M. ulcerans* DNA from environmental and animal samples. Data extracted from publications on environmental detection of *M. ulcerans* included: i) sample dates and location, ii) sample type (e.g. water, soil, plant, animal [faeces/clinical]), iii) taxonomic details for animal samples, iv) confirmatory tests applied (including all PCR targets tested) and their results, and v) number of samples tested and positive.
We additionally identified locations reported to be absent of or non-endemic for BU.
For these studies we identified study dates, location, and case-ascertainment strategy (e.g. passive case detection, active case search, cross-sectional survey).

S.1.2. Remote georeferencing details

Geographical information extracted from publications included geographical coordinates, site name, upper administrative unit, and any other contextual information.

Remote geo-referencing was used to link results to the study location if the coordinates were not given. If a fine scale map of occurrence locations was provided, this was converted to a raster format and georeferenced in ArcGIS Desktop 10.5 (Environmental Systems Research Institute Inc., Redlands CA, USA) to allow extraction of approximate geographic coordinates of the study locations. Locations given as community names were georeferenced using online geodatabases. Automated georeferencing was implemented in R software through the Google Maps API Engine and Opencage Geocoder, using the ggmap and opencage packages respectively (1, 2). Locations that could not be georeferenced by these methods were manually searched in Google.

An ordinal score was assigned to reflect the reliability of the georeferenced locations (Table S.1).

Georeferenced points were mapped using ArcGIS Desktop 10.5 (Environmental Systems Research Institute Inc., Redlands CA, USA). Points falling outside of a land boundary or outside of the upper administrative unit they we assigned to in the publication were checked, and if a more reliable match could not be found, excluded.

S.1.3 Data quality score for occurrence records of BU disease in humans

All occurrence records from national programme data and selected publications were assigned local- and national-level evidence quality scores reflecting the contemporariness and specificity of the diagnosis.

Records of cases diagnosed since 2003 were given a contemporariness score of 1. Records of cases from 1990- 2002 were down-weighted by 50% and those from before 1990 were down weighted by 75%, under the assumption that historically endemic areas may no longer be endemic.

The diagnostic specificity score ranged from 0.5 to 1. The maximum score for was given for records where confirmation by PCR indicating *M. ulcerans*, or histopathological analysis indicating BU disease was reported. Although the WHO case definition for a confirmed case requires at least two positive confirmatory tests (3), we decided not to down-weight cases with only one confirmatory test result because multiple testing is uncommon due to the higher resource requirements, and comparative analysis of different confirmatory testing methods has shown the common tests to have a high specificity when used individually (4). However, records where cases were confirmed by Ziehl Neelsen (ZN) staining and/or culture alone were down-weighted by 5%, reflecting the possibility of detection of other mycobacteria by these methods (5, 6). The score was down-weighted by 50% if cases were clinically diagnosed only. If the publication reported that proportion of cases were laboratory-confirmed, but did not present the confirmation status of cases at unique locations, the record was considered unconfirmed at local-level, and confirmed at the national-level.

The contemporariness and diagnosis scores were summed to provide local- and national-level evidence quality scores for each occurrence record. Local evidence quality scores were converted to percentages, which were used as weights to adjust the total number of cases reported at each location.

For countries from which strains of *M. ulcerans* were reported to have been isolated, but with no evidence of reported cases meeting the inclusion criteria, the evidence score was adjusted *post-hoc*.

S.1.4. Data quality score for environmental detection of M. ulcerans DNA and BU disease in animals

Various PCR targeting techniques with varying sensitivities and specificities are used for *M. ulcerans* detection. The conventional PCR target for confirmation of human cases is the IS (insertion sequence) *2404*, present at high copy number in the *M. ulcerans* genome (7), and providing high sensitivity and specificity (8, 9). This target can also be used to indicate probable presence of *M. ulcerans* in the environment, but its sensitivity and specificity are reduced by the existence of PCR inhibitors and of other mycobacteria carrying the same gene within environmental samples. Other PCR targets available include the IS*2606*, and sequences encoding the enoyl reductase (ER) and ketoreductase-B (KR-B) domains, which form part of the mycolactane polyketide synthase genes [ref]. These genes are also present in other mycolactane-producing mycobacteria (MPM), but at different copy numbers (7). Recently developed multiplex qPCR assays targeting IS*2404*, IS*2606* and the KR-B domain and quantifying their respective copy numbers allow discrimination of *M. ulcerans* from other MPM (7). Variable nucleotide tandem repeat (VNTR) and mycobacterial interspersed repetitive unit (MIRU) typing can also be used to distinguish *M. ulcerans* from other MPM (10) based on the copy numbers of short repeating DNA sequences found at multiple loci within the genome (11).

According to the varying discriminatory power of these typing methods, we assigned the maximum typing score to samples confirmed as *M. ulcerans* by multiplex qPCR or VNTR analysis, a lower

score for samples typed only by conventional PCR analysis targeting IS*2404*, IS*2606*, ER or KR-B, alone or in combination. Samples confirmed as MPM other than *M. ulcerans* (by qPCR or VNTR typing) were down-weighted by 75% relative to those confirmed as *M. ulcerans*, on the assumption that these organisms may share a similar niche to *M. ulcerans*, and indeed have been considered by some authors to be part of the same species (12).

Each environmental/animal occurrence record was given a data quality score from 0-3 based on:

Typing specificity (0-2):

- confirmed as MPM other than *M. ulcerans* = 0.5
- typed only by conventional PCR analysis targeting IS2404, IS2606, ER or KR-B, alone or in combination = 1
- confirmed as *M. ulcerans* by multiplex qPCR or VNTR analysis on environmental samples (including animal faeces) or by clinical diagnosis and positive result for IS2404 PCR = 2

Contemporariness (0 -1)

- Prior to 1990 = 0.25
- 1990- 2002 = 0.5
- 2003- 2018 = 1

Each upper administrative unit {Global Administrative Areas, 2012 #14621} was assigned the highest data quality score of all environmental/ animal occurrence records within it, and a score reflecting the total number of environmental/ animal occurrence records within it

Number of occurrences (0 -1)

- 4 = 0.25
- 4- 10 = 0.5
- 10- 20 = 0.75
- >20 = 1

Within each administrative unit, the scores were summed to give a score from 0- 4, and converted to a percentage for mapping.

S.2. Evidence consensus framework

An evidence consensus framework was used to assign scores reflecting the strength of evidence for BU presence and absence at national level. A separate framework was used to assess the strength of evidence for BU presence at the highest sub-national administrative level (adm1) within countries with evidence of BU presence.

For countries with reported evidence of BU cases, four main data sources were used for the evidence consensus: i) BU data reported nationally to WHO from 2007- 2016, ii) reports of BU disease to GIDEON, iii) reports of cases in peer-reviewed literature, and iv) cases recorded by surveillance programmes or public health laboratories in BU-endemic countries. These were converted to three constituent scores: health organisation status, occurrence data quality, and case number.

The occurrence data quality score was based on the highest national data quality score assigned to each occurrence record. Countries with no occurrence data were assigned a score of 0, and those with occurrence data were assigned a score of up to 3 (if cases were recorded since 2003 and laboratory-confirmed).

The case number score was based on the total number of cases in all occurrence records, each adjusted by its local-level evidence quality score. Countries reporting more than 20 cases (post-adjustment) were given a score of 1, those reporting 11-20 were down-weighted by 25%, those reporting 4- 10 were down-weighted by 50%, and those reporting fewer than 4 cases were down-weighted by 75%.

Consensus presence was assigned if cases had been reported to WHO between 2002 and 2018; BU had been reported through GIDEON; at least one laboratory confirmed case had been recorded in peer-reviewed literature or by the national programme (for countries which had contributed surveillance data); and if a minimum number had been reported from all sources- the minimum threshold ranged from 20, if all cases were laboratory confirmed and reported since 2003, and was scaled up depending on the proportions unconfirmed and reported prior to 2003.

If there was no evidence of BU from any of the data sources included, the evidence consensus score was designed to quantify the strength of evidence for BU absence, reflecting the possibility of cases being under-reported due to weak surveillance or reporting capacity, or being masked due to misdiagnosis as known endemic diseases that share diagnostic presentations with BU (potential confounding diseases).

The potential confounding diseases with evidence available on their global distribution were cutaneous leishmaniasis (CL), leprosy, lymphatic filariasis (LF), onchocerciasis and tropical ulcer (TU), all of which have at least one possible presentation in common with BU (including nodules, plaques, oedema and ulcers) (13). The country-level endemicity of these diseases was based on

evidence consensus mapping for CL (14, 15); literature review for yaws (16), GIDEON data for TU (17); the rapid epidemiological mapping of onchocerciasis (REMO) (18); and on reporting of leprosy to WHO from 2012- 2016 (data provided on request by the WHO Leprosy team).

Estimates of the proportional frequencies of the most common presentations of BU and the potential confounding diseases were obtained from literature review (18-22) and expert opinion, using cross-sectional survey data preferentially to health facility data, as the latter would tend to overestimate the proportion of cases with more severe presentations. Prevalence of onchocerciasis in the REMO study was based only on nodule prevalence, so the frequency of nodules among onchocerciasis cases was set at 100%.

For each disease, the proportional frequencies of the presentations shared with BU were multiplied by the proportional frequency of the corresponding presentation in BU cases, and the products summed to generate a symptom overlap score, reflecting the likelihood of misdiagnosis of BU as that disease (Supplementary Figure S1, Table S.1). For each country, misdiagnosis likelihood scores for all endemic confounding diseases were summed and standardised to a percentage, representing a composite misdiagnosis likelihood score.

Health expenditure values (HE; average expenditure from 2011- 2015, from all financing sources, expressed in constant (2010) USD per capita) reported by the WHO (23) were used as a proxy for diagnostic capacity and for surveillance and reporting capacity, following the approach of previous authors (15, 24, 25) and supported by evidence that a higher level of health expenditure is associated with better health system performance (26). Countries with HE <$100 were categorised as low, those with $100≤HE<$500 were categorised as medium, and those with HE≥$500 were categorised as high.

Two separate health system scores were assigned based on HE category. The first was used to adjust composite misdiagnosis likelihood scores, reflecting lower likelihood of misdiagnosis of BU in countries with higher health expenditure, assuming a higher diagnostic capacity. The final composite misdiagnosis likelihood score was intended to indicate the likelihood of BU being misdiagnosed as any of the confounding diseases in each country. HE was also used to assign a score representing surveillance and reporting capacity. Countries with low HE were considered most likely to be under-detecting or not reporting BU so were assigned a score of 1, while those with high HE were assigned a score of 0.

Consensus absence was assigned to countries with no evidence of BU cases reported through WHO or GIDEON, or in peer-reviewed literature, no evidence of endemicity of the potential confounding diseases considered, and high health expenditure. Countries endemic for all the confounding diseases and with low HE scored 0, reflecting indeterminate BU endemicity status.

S.3. Quality assessment framework for BU prevalence surveys

This framework is adapted from Deribe et al. (27), and grades surveys on an 8 point scale using 4 quality assessment elements:

A. Definition of sampling frame

0. No information beyond overall population type (e.g. “schools” or “households”)

1. General information on sampling frame and procedures

2. Explicit details of procedures reported

B. Survey coverage

0. Not recorded/reported

1. Reported and under 65%

2. Reported and 65% or above

C. Specificity of BU diagnosis

0. No detail on diagnosis, or clinical diagnosis reported but not adequately described

1. Clinical diagnosis adequately described (e.g. WHO guidelines, or justified modification of standard diagnostic criteria)

2. Clinical diagnosis with all or a subset confirmed by laboratory tests

D. Statistical analysis

0. Only overall prevalence reported

1. Prevalence reported with 95% CIs

2. Prevalence by subgroup (e.g. age, sex, socio-demographic) reported

References for supplementary methods

1. Kahle D. and Wickham H. ggmap: Spatial Visualization with ggplot2. *The R Journal*. 2013;**5**(1):144- 61
2. Salmon M. opencage: Interface to the OpenCage API. R package version 0.1.2. 2017
3. World Health Organisation. Buruli ulcer - Diagnosis of Mycobacterium ulcerans disease [Available from: http://www.who.int/buruli/information/diagnosis/en/index2.html].
4. Bretzel G, Siegmund V, Nitschke J, et al. A stepwise approach to the laboratory diagnosis of Buruli ulcer disease. *Tropical medicine & international health : TM & IH*. 2007;**12**(1):89-96
5. Bratschi MW, Tabah EN, Bolz M, et al. A Case of Cutaneous Tuberculosis in a Buruli Ulcer-Endemic Area. *PLoS neglected tropical diseases*. 2012;**6**(8)
6. Debacker M, Aguiar J, Steunou C, et al. Mycobacterium ulcerans disease (Buruli ulcer) in rural hospital, Southern Benin, 1997–2001. *Emerging infectious diseases*. 2004;**10**(8):1391
7. Fyfe JAM, Lavender CJ, Johnson PDR, et al. Development and application of two multiplex real-time PCR assays for the detection of Mycobacterium ulcerans in clinical and environmental samples. *Applied and environmental microbiology*. 2007;**73**(15):4733-40
8. Sakyi SA, Aboagye SY, Darko Otchere I, et al. Clinical and Laboratory Diagnosis of Buruli Ulcer Disease: A Systematic Review. *The Canadian journal of infectious diseases & medical microbiology = Journal canadien des maladies infectieuses et de la microbiologie medicale*. 2016;**2016**:5310718
9. Stinear T, Davies JK, Jenkin GA, et al. A simple PCR method for rapid genotype analysis of Mycobacterium ulcerans. *Journal of clinical microbiology*. 2000;**38**(4):1482-7
10. Lavender CJ, Stinear TP, Johnson PDR, et al. Evaluation of VNTR typing for the identification of Mycobacterium ulcerans in environmental samples from Victoria, Australia. *FEMS Microbiol Lett*. 2008;**287**(2):250-5
11. Stragier P, Ablordey A, Meyers WM, et al. Genotyping Mycobacterium ulcerans and Mycobacterium marinum by using mycobacterial interspersed repetitive units. *J Bacteriol*. 2005;**187**(5):1639-47
12. Pidot SJ, Asiedu K, Kaser M, et al. Mycobacterium ulcerans and Other Mycolactone-Producing Mycobacteria Should Be Considered a Single Species. *PLoS neglected tropical diseases*. 2010;**4**(7)
13. World Health Organisation. Recognizing Neglected Tropical Diseases through Changes on The Skin. Compiled 2018.
14. Pigott DM, Bhatt S, Golding N, et al. Global distribution maps of the leishmaniases. *eLife*. 2014;**3**
15. Pigott DM, Bhatt S, Golding N, et al. Data from: Global distribution maps of the Leishmaniases. Compiled: Dryad Data Repository; 2014.
16. Mitja O, Marks M, Konan DJ, et al. Global epidemiology of yaws: a systematic review. *The Lancet Global health*. 2015;**3**(6):e324-31
17. Berger S. Tropical Skin Ulcers: Global Status. Gideon Informatics Inc., editor2018.
18. Zouré HG, Noma M, Tekle AH, et al. The geographic distribution of onchocerciasis in the 20 participating countries of the African Programme for Onchocerciasis Control:(2) pre-control endemicity levels and estimated number infected. *Parasites & vectors*. 2014;**7**(1):326
19. Marks M, Chi K-H, Vahi V, et al. - Haemophilus ducreyi associated with skin ulcers among children, Solomon Islands. 2014;**- 20**(- 10):- 1707
20. Coldiron M, Obvala D, Mouniaman-Nara I, et al. The prevalence of yaws among the Aka in the Congo. *Medecine et sante tropicales*. 2013;**23**(2):231-2
21. Remadi L, Haouas N, Chaara D, et al. Clinical presentation of cutaneous leishmaniasis caused by Leishmania major. *Dermatology*. 2016;**232**(6):752-9
22. Mwingira UJ, Downs P, Uisso C, et al. Applying a mobile survey tool for assessing lymphatic filariasis morbidity in Mtwara Municipal Council of Tanzania. *mHealth*. 2017;**3**
23. World Health Organisation. Global Health Expenditure Database. Compiled.
24. Brady OJ, Gething PW, Bhatt S, et al. Refining the global spatial limits of dengue virus transmission by evidence-based consensus. *PLoS neglected tropical diseases*. 2012;**6**(8):e1760

25. Deribe K, Cano J, Newport MJ, et al. The global atlas of podoconiosis. *The Lancet Global Health*. 2017;**5**(5):e477-e9
26. Evans DB, Tandon A, Murray CJ, et al. Comparative efficiency of national health systems: cross national econometric analysis. *BMJ (Clinical research ed)*. 2001;**323**(7308):307-10
27. Deribe K, Cano J, Trueba ML, et al. Global epidemiology of podoconiosis: A systematic review. *PLoS neglected tropical diseases*. 2018;**12**(3):e0006324

Supplementary Results

Table S.1: Frequencies of shared presentations of BU and other skin diseases used to calculate symptom overlap scores for confounding diseases

**Confounding disease** *Reference*	**Common presentations**		**Buruli Ulcer** *Ampah et al 2016 (1); Johnson et al 2005 (2); um Boock et al 2004 (3) (pooled)*			
			Nodule	Plaque	Oedema	Ulcer
		**p~ with presentation**	0·057	0·007	0·025	0·709
**Yaws** *Coldiron et al. 2013 (4)*	Crusted ulcer	0·23				**0·1631**
**Cutaneous leishmaniasis** *Remadi et al. 2016 (5)*	Ulcero-crusted	0·39				**0·2744**
	Ulcerated	0·11				**0·0759**
**Onchocerciasis** *Zouré, 2014 (6)*	Nodules	1·00	**0·0567**			
**Lymphatic filariasis** *Mwingira 2017 (7)*	Oedema	0·209			**0·0052**	
**Leprosy** *P. Saunderson, personal communication, June 2018 (8)*	Plaques	0·05		**0·0003**		
	Ulcers	0·05				**0·0355**
**Tropical Ulcer** *Berger 2018 (9)*	Ulcers	1·000				**0·7091**

References for Table S.1

1. Ampah KA, Asare P, Binnah DD, et al. Burden and Historical Trend of Buruli Ulcer Prevalence in Selected Communities along the Offin River of Ghana. *PLoS neglected tropical diseases*. 2016;**10**(4):e0004603
2. Johnson RC, Sopoh GE, Boko M, et al. [Distribution of Mycobacterium ulcerans (Buruli ulcer) in the district of Lalo in Benin]. *Tropical medicine & international health : TM & IH*. 2005;**10**(9):863-71
3. Um Boock A. Enquete Preliminaire Nationale sur la Situation de l'Ulcere de Buruli au Cameroun. 2004
4. Coldiron M, Obvala D, Mouniaman-Nara I, et al. The prevalence of yaws among the Aka in the Congo. *Medecine et sante tropicales*. 2013;**23**(2):231-2
5. Remadi L, Haouas N, Chaara D, et al. Clinical presentation of cutaneous leishmaniasis caused by Leishmania major. *Dermatology*. 2016;**232**(6):752-9
6. Zouré HG, Noma M, Tekle AH, et al. The geographic distribution of onchocerciasis in the 20 participating countries of the African Programme for Onchocerciasis Control:(2) pre-control endemicity levels and estimated number infected. *Parasites & vectors*. 2014;**7**(1):326
7. Mwingira UJ, Downs P, Uisso C, et al. Applying a mobile survey tool for assessing lymphatic filariasis morbidity in Mtwara Municipal Council of Tanzania. *mHealth*. 2017;**3**
8. Saunderson P. Personal communication. Compiled 2018.
9. Berger S. Tropical Skin Ulcers: Global Status. Gideon Informatics Inc., 2018.

Table S.2: Scores assigned based on the number of human cases at national level and by upper administrative unit, and on positive environmental samples by upper administrative unit.

Number of human cases/ positive environmental samples	Score
1- 3	0.25
4- 10	0.5
11- 20	0.75
>20	1

Figure S.1: Number of references identified in literature review, reported case confirmation methods, and number of cases reported to WHO 2007-2016 by country

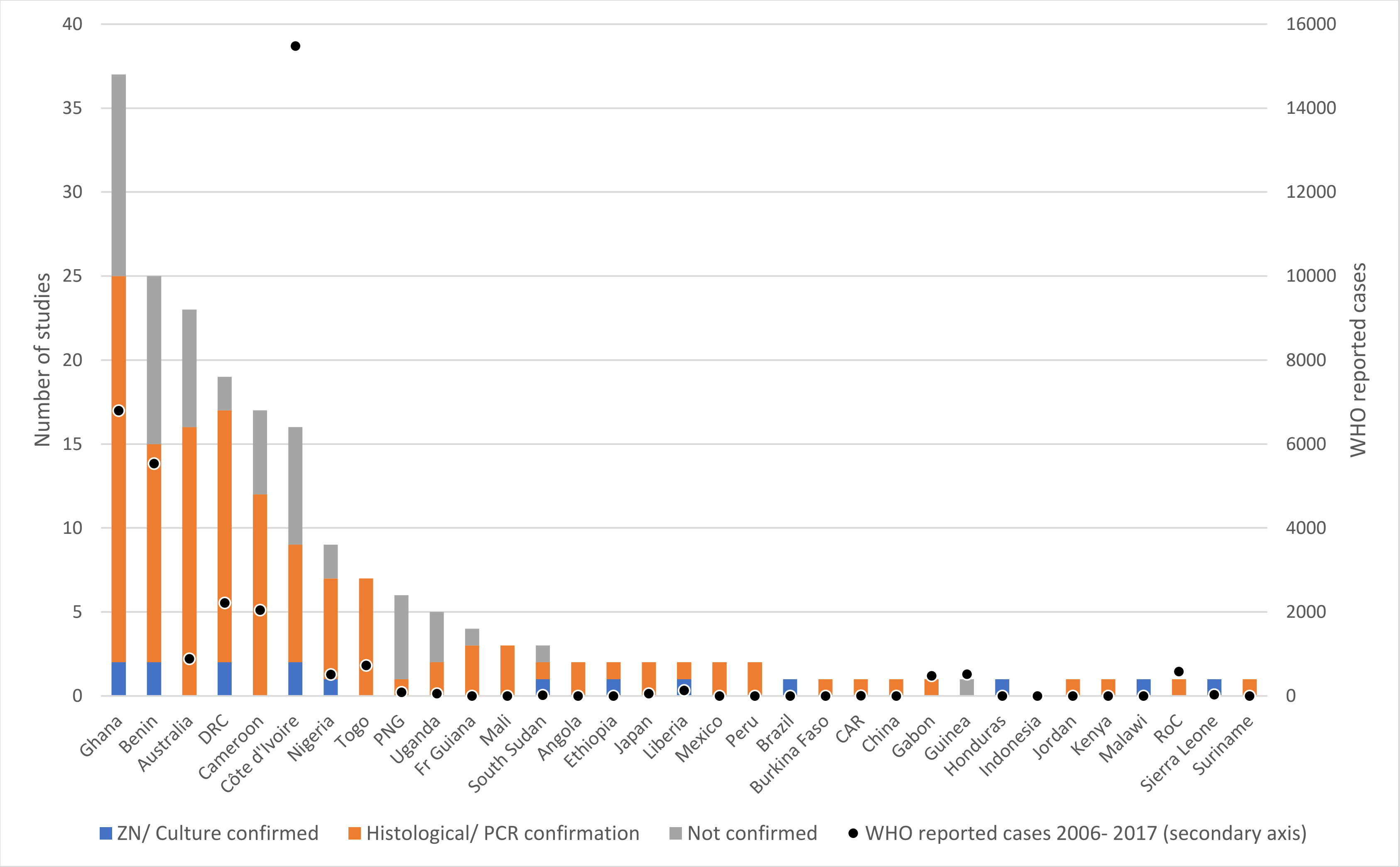

DRC = Democratic Republic of Congo, PNG = Papua New Guinea, CAR = Central African Republic

Figure S.2: Evidence consensus maps showing the distribution of occurrence points identified by systematic literature search.

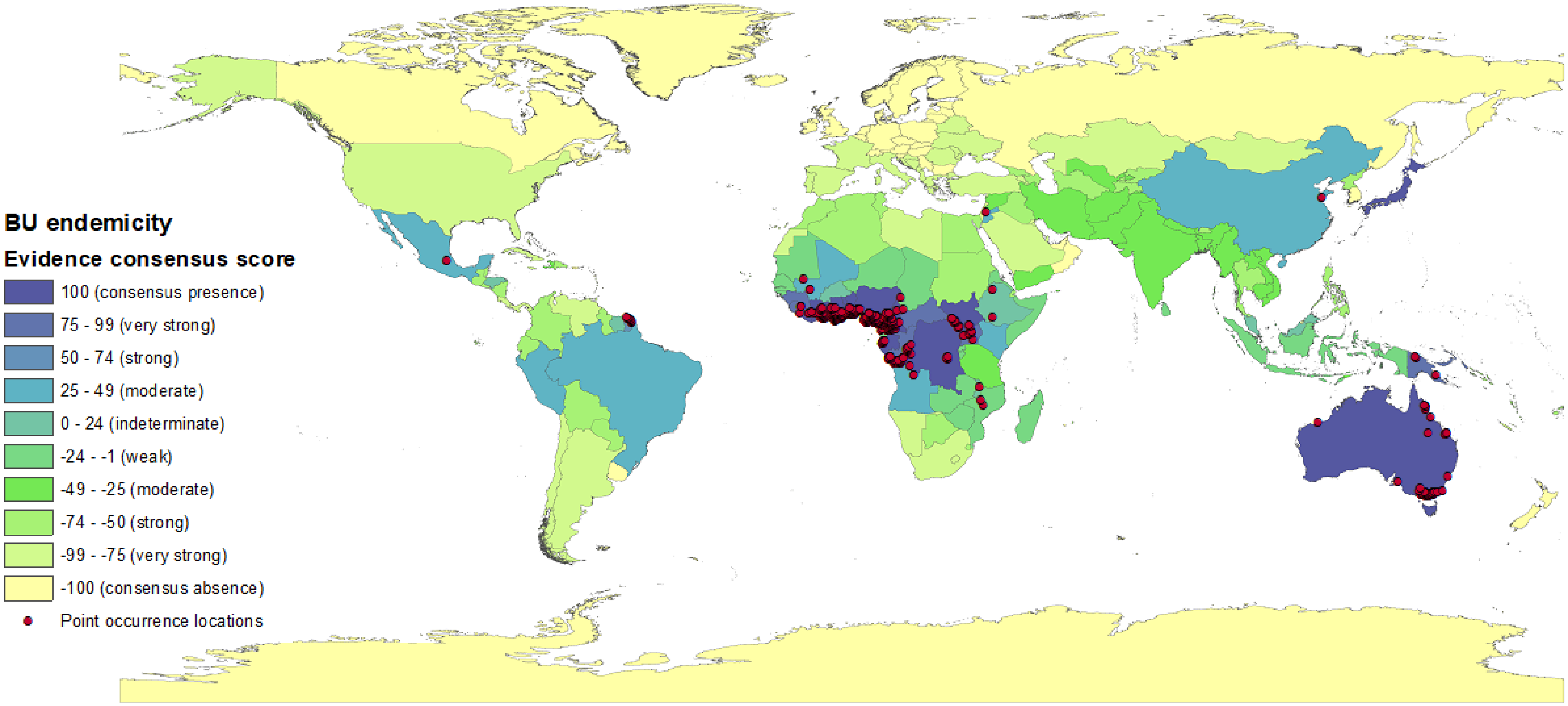

Table S.3: Data extracted from selected studies used for evidence consensus framework- human cases

Country	Reference		Recording	Confirmation method				Scores assigned			Number of cases	
	Main author (year published)	Ref	year	ZN	Culture	Histological	PCR	Diagnosis	Contemporariness	Quality	Included	Adjusted
**Angola**	Kibadi, K. et al. (2008)	(1)	2005	Yes			Yes	1	1	1	2	2
	Bar, W. et al. (1998)	(2)	1995	Yes		Yes	Yes	1	0.5	0.75	1	0.75
**Australia**	Tai, A. et al. (2018)	(3)	2017	Yes	Yes	Yes	Yes	1	1	1	434	423.15
	Boyd, S. et al. (2012)	(4)	2011	Yes			Yes	1	1	1	180	180
	Lavender, C. et al. (2011)	(5)	2009				Yes	1	1	1	81	81
	Quek, T. et al. (2007 a)	(6)	2006	Yes			Yes	1	1	1	76	76
	Quek, T. et al. (2007 b)	(7)	2005	Yes			Yes	1	1	1	42	42
	Steffen, C. et al. (2010)	(8)	2008	Yes		Yes	Yes	1	1	1	41	40.925
	WHO (Johnson) (2006)	(9)	2005		Yes		Yes	1	1	1	40	40
	O'Brien, D. et al. (2007)	(10)	2004	Yes		Yes	Yes	1	1	1	40	40
	Johnson, P. et al. (2007)	(11)	2006		Yes		Yes	1	1	1	31	29.25
	Carson, C. et al. (2014)	(12)	2013		Yes		Yes	1	1	1	6	6
	Francis, G. et al. (2006)	(13)	2009			Yes	Yes	1	1	1	4	4
	van Ravensway, J. et al. (2012)	(14)	2008					0.5	1	0.75	87	65.25
	Johnson, P. et al. (1996)	(15)	1995	Yes	Yes	Yes		1	0.5	0.75	42	28.6
	Veitch, M. et al. (1997)	(16)	1995			Yes		1	0.5	0.75	28	21
	Gooding, T. et al. (2002)	(17)	2002				Yes	1	0.5	0.75	23	17.25
	O'Brien, D. et al. (2017)	(18)	2014				Yes	0.5	1	0.75	20	16.5
	WHO (Johnson) (2001)	(19)	2001		Yes		Yes	1	0.5	0.75	14	7
	Fyfe, J. et al. (2010)	(20)	2009					0.5	1	0.75	4	3
	Taheri, T. et al. (2009)	(21)	2009					0.5	1	0.75	1	0.75
	WHO (Johnson) (2000)	(22)	1999					0.5	0.5	0.5	8	4
	WHO (2003)	(23)	2002					0.5	0.5	0.5	1	0.5
	Mitchell, P. et al. (1987)	(24)	1985					0.5	0.25	0.375	20	7.5
	Stinear, T. et al. (2000)	(25)	1987					0.5	0.25	0.375	11	4.125
**Benin**	Amoussouhoui, A. et al. (2018)	(26)	2016				Yes	1	1	1	137	102.75
	Sopoh, G. et al. (2010)	(27)	2008	Yes	Yes		Yes	1	1	1	104	101.4
	Nackers, F. et al. (2006)	(28)	2003	Yes	Yes	Yes	Yes	1	1	1	70	52.5
	Eddyani, M. et al. (2015)	(29)	2008				Yes	1	1	1	46	46
	Ruf, M. T. et al. (2011)	(30)	2009	Yes	Yes	Yes	Yes	1	1	1	12	12
	Andreoli, A. et al. (2014)	(31)	2009	Yes		Yes	Yes	1	1	1	8	8
	Barogui, Y. T. et al. (2018)	(32)	2016				Yes	1	1	1	6	6
**Brazil**	Leigheb, G. et al. (2008)	(33)	2006	Yes	Yes	Yes	Yes	1	1	1	2	2
	Sopoh, G. et al. (2010)	(34)	2007	Yes		Yes	Yes	1	1	1	1	1
	Wadagni, A. et al. (2018)	(35)	2016					0.5	1	0.75	6908	5181
	Debacker, M. et al. (2004)	(36)	2001	Yes	Yes	Yes	Yes	1	0.5	0.75	1131	848.25
	Johnson, R. et al. (2015)	(37)	2012					0.5	1	0.75	861	645.75
	Campbell, L. et al. (2015)	(38)	2005					0.5	1	0.75	558	418.5
	Sopoh, G. et al. (2010)	(39)	2006					0.5	1	0.75	425	318.75
	WHO (2001) (Guédénon)	(19)	2000	Yes	Yes	Yes	Yes	1	0.5	0.75	410	307.5
	Barogui, Y. et al. (2009)	(40)	2006					0.5	1	0.75	160	120
	Johnson, P. et al. (2005)	(41)	2004					0.5	1	0.75	160	119.25
	Sopoh, G. et al. (2011)	(42)	2009					0.5	1	0.75	125	93.75
	Durnez, L. et al. (2010)	(43)	2006					0.5	1	0.75	46	34.5

Country	Reference		Recording	Confirmation method				Scores assigned			Number of cases	
	Main author (year published)	Ref	year	ZN	Culture	Histological	PCR	Diagnosis	Contemporariness	Quality	Included	Adjusted
	Williamson, H. et al. (2012)	(44)	2009					0.5	1	0.75	12	9
	Ablordey, A. et al. (2015)	(45)	2000				Yes	1	0.5	0.75	3	2.25
	Abalos, F. et al. (2000)	(46)	2000	Yes	Yes	Yes	Yes	1	0.5	0.75	1	0.75
	Josse, R., et al. (1994)	(47)	1993	Yes				0.95	0.5	0.725	225	163.125
	Josse, R., et al. (2002)	(48)	1990	Yes				1	0.25	0.625	45	27
	Stoffel, V. et al. (2005)	(49)	2002					0.5	0.5	0.5	15	7.5
	dos Santos, J. (2007)	(50)	2004		Yes			0.95	1	0.975	1	0.975
**Burkina Faso**	Ouoba, K., et al. (1998)	(51)	1996	Yes	Yes	Yes		1	0.5	0.75	4	2.25
**Cameroon**	Cameroon MoH (2017)	(52)	2015	Yes			Yes	1	1	1	2774	2768.8
	Christenet, V (2014) [1]	(53)	2012	Yes	Yes	Yes	Yes	1	1	1	1113	1083.475
	Landier, J. et al. (2011)	(54)	2009			Yes	Yes	1	1	1	171	171
	Bratschi, M. et al. (2013)	(55)	2012				Yes	1	1	1	148	131.25
	Marion, E. et al. (2011)	(56)	2009				Yes	1	1	1	125	111.5
	Porten, K. et al. (2009)	(57)	2007					1	1	1	105	78.75
	Bratschi, M. et al. (2014)	(58)	2011	Yes			Yes	1	1	1	57	57
	Zambou, M. et al. (2011)	(59)	2010	Yes			Yes	1	1	1	54	54
	Bolz, M. et al. (2015)	(60)	2012				Yes	1	1	1	39	39
	Awah, P. K. et al. (2018)	(61)	2014					1	1	1	32	24
	Wanda, F. et al. (2014)	(62)	2013	Yes		Yes	Yes	1	1	1	1	1
	Andreoli, A. et al. (2015)	(63)	2011	Yes			Yes	1	1	1	1	1
	Landier, J. et al. (2014)	(64)	2012	Yes			Yes	0.95	1	0.975	588	541.05
	Um Boock A. (2004)	(65)	2004					0.5	1	0.75	123	92.25
	Zogo, B. et al. (2015)	(66)	2013					0.5	1	0.75	97	72.75
	Noeske, J. et al. (2004)	(67)	2001	Yes			Yes	1	0.5	0.75	68	34
	Ebong, S. M. et al. (2012)	(68)	2012					0.5	1	0.75	16	12
**CAR**	Minime-Lingoupou, F. et al. (2010)	(69)	2007	Yes			Yes	1	1	1	2	2
**China**	Faber, W. R. et al. (2000)	(70)	2000	Yes			Yes	1	0.5	0.75	1	0.75
**Côte d'Ivoire**	N'Krumah, R. et al. (2017)	(71)	2010				Yes	1	1	1	1145	1145
	N'Krumah R, T. et al. (2016)	(72)	2012				Yes	1	1	1	51	51
	Coulibaly-N'Golo, G. et al. (2011)	(73)	2008				Yes	1	1	1	14	14
	Kouame, K. et al. (2008)	(74)	2007	Yes		Yes		1	1	1	4	3.9
	Sangare, A. (2007)	(75)	2005	Yes		Yes		1	1	1	1	1
	Ahoua et al (2009)	(76)	2009		Yes		Yes	1	1	0.975	116	96.125
	Ecra, E., et al. (2005)	(77)	2000	Yes		Yes		1	0.5	0.75	754	390.5
	Konan, K. L. et al. (2015)	(78)	2007					0.5	1	0.75	11	8.25
	Boni, C. C., et al. (2017)	(79)	2014					0.5	1	0.75	5	3.75
	Darie, H. (1993)	(80)	1992	Yes				1	0.5	0.75	4	3
	Ablordey, A. et al. (2015)	(45)	2000				Yes	1	0.5	0.75	2	1.5
	Richard Kadio (1990)	(81)	1990	Yes				0.95	0.25	0.6	167	62.625
	Kanga et al. (2001)	(82)	1997					0.5	0.5	0.5	2409	1204.5
	WHO (2001) (Kanga)	(19)	1999					0.5	0.5	0.5	1351	675.5
	Ecra, E. J., et al. (2001)	(83)	2001					0.5	0.5	0.5	2	1
	Espey, David K. et al. (2002)	(84)	1994					0.5	0.5	0.5	2	1
	Marston, B. et al. (1995)	(85)	1991					0.5	0.5	0.5	2	1
**DRC**	Mavinga Phanzu et al. (2013)	(86)	2008	Yes		Yes	Yes	1	1	1	259	194.25
	Mavinga Phanzu, D. et al. (2011)	(87)	2007	Yes	Yes	Yes	Yes	1	1	1	252	189

Country	Reference		Recording	Confirmation method				Scores assigned			Number of cases	
	Main author (year published)	Ref	year	ZN	Culture	Histological	PCR	Diagnosis	Contemporariness	Quality	Included	Adjusted
	Kibadi, K. et al. (2010)	(88)	2007	Yes	Yes	Yes	Yes	1	1	1	92	92
	Suykerbuyk, P. et al. (2009)	(89)	2007				Yes	1	1	1	28	28
	Mavinga Phanzu, D. et al. (2006)	(90)	2004	Yes	Yes	Yes	Yes	1	1	1	36	27
	Kibadi, K. et al. (2009)	(91)	2007				Yes	1	1	1	19	19
	Mavinga Phanzu, D. et al. (2011)	(92)	2017	Yes	Yes	Yes	Yes	1	1	1	13	13
	Kibadi, K. et al. (2008)	(1)	2005	Yes		Yes	Yes	1	1	1	1	1
	Kibadi, K. (2009)	(93)	2007					0.5	1	0.75	1	0.75
	Mavinga Phanzu, D. et al. (2007)	(94)	2002	Yes		Yes	Yes	1	0.5	0.75	1	0.75
	Delaporte E. et al. (1994)	(95)	1992			Yes		1	0.5	0.75	1	0.75
	Meyers, M. W. (1974)	(96)	1972	Yes		Yes		1	0.25	0.625	78	33
	Smith, J. H. (1970)	(97)	1968	Yes	Yes	Yes		1	0.25	0.625	3	1.8
	Ablordey, A. et al. (2015)	(45)	1971				Yes	1	0.25	0.625	1	0.625
	Andersen, F. O. (1965)	(98)	1964	Yes		Yes		1	0.25	0.625	1	0.625
	Meyers, M. W. (1975)	(99)	1973	Yes		Yes		0.95	0.25	0.6	180	108
	Guerden, A. (1962)	(100)	1962	Yes				0.95	0.25	0.6	170	102
	Pattyn, S. R. (1965)	(101)	1965					0.95	0.25	0.6	1	0.6
	Hennebert, P. et al, (1962)	(102)	1960	Yes				0.95	0.25	0.6	1	0.6
**Ethiopia**	Gordon, D. et al. (2014)	(103)	2011	Yes				0.95	1	0.975	1	0.975
	Haileamlak A. et al. (2009)	(104)	2009			Yes		0.5	1	0.75	1	0.75
**French Guiana**	Reynaud, Y. et al. (2015)	(105)	2013				Yes	1	1	1	23	23
	Douine, M. (2017)	(106)	2013	Yes	Yes	Yes	Yes	1	1	1	12	6.5
	Ménard, A. et al. (2003)	(107)	1999	Yes	Yes	Yes	Yes	1	0.5	0.75	3	2.25
	WHO (2003)	(23)	2001					0.5	0.5	0.5	7	3.5
**Gabon**	Bayonne Manou, L. S. et al. (2013)	(108)	2011	Yes	Yes		Yes	1	1	1	300	294.725
**Ghana**	Yeboah-Manu, D. et al. (2018)	(109)	2016	Yes	Yes		Yes	1	1	1	1020	1020
	Ghana MOH (KCCR) (2017)	(110)	2039				Yes	1	1	1	628	628
	Ghana MoH (NBUCP) (2015)	(111)	2014	Yes			Yes	1	1	1	393	361.725
	Amissah, N. et al. (2014)	(112)	2011				Yes	1	1	1	245	245
	Sarfo, F. S. et al. (2010)	(113)	2007	Yes	Yes		Yes	1	1	1	160	160
	Bibert, S. et al. (2017)	(114)	2017				Yes	1	1	1	96	96
	Adu, E. J. (2013)	(115)	2012	Yes		Yes	Yes	1	1	1	65	63.375
	Roltgen, K. et al. (2010)	(116)	2006				Yes	1	1	1	63	63
	Phillips, R. O. et al. (2009)	(117)	2007	Yes			Yes	1	1	1	45	44.5
	Aboagye, S. et al. (2017)	(118)	2014				Yes	1	1	1	32	32
	Sarfo, F. S. et al. (2011)	(119)	2011	Yes	Yes		Yes	1	1	1	26	26
	Ablordey, A. et al. (2015)	(45)	2012				Yes	1	1	1	24	22.75
	Amissah, N. et al. (2015)	(120)	2013				Yes	1	1	1	19	19
	Yeboah-Manu, D. et al. (2012)	(121)	2012				Yes	1	1	1	21	16
	Narh, C. A. et al. (2015)	(122)	2015				Yes	1	1	1	14	14
	Ampah, K. A. et al. (2016)	(123)	2013				Yes	1	1	1	12	12
	Iddrisah, F. et al. (2016)	(124)	2010				Yes	1	1	1	2	2
	Sarfo, F. S. et al. (2014)	(125)	2011				Yes	1	1	1	1	1
	Hospers, I. C. et al. (2005)	(126)	2002			Yes		1	0.5	0.75	396	297
	Wu, J. et al. (2015)	(127)	2010					0.5	1	0.75	326	244.5
	Ackumey, M. M. et al. (2011)	(128)	2008					0.5	1	0.75	297	222.75

Country	Reference		Recording	Confirmation method				Scores assigned			Number of cases	
	Main author (year published)	Ref	year	ZN	Culture	Histological	PCR	Diagnosis	Contemporariness	Quality	Included	Adjusted
	Kenu, E. et al. (2014)	(129)	2011					0.5	1	0.75	118	88.5
	Raghunathan, P. L. et al. (2005)	(130)	2000	Yes	Yes	Yes	Yes	1	0.5	0.75	93	69.75
	Hamzat et al. (2011)	(131)	2011					0.5	1	0.75	84	63
	Kotey, N. K. and Ampadu, X. (2011)	(132)	2012	Yes	Yes		Yes	0.5	1	0.75	68	51
	Osei-Sarpong, F. (2015)	(133)	2005					0.5	1	0.75	62	46.5
	Tschakert, P. et al. (2016)	(134)	2009					0.5	1	0.75	47	35.25
	Williamson, H. R. et al. (2008)	(135)	2006					0.5	1	0.75	36	27
	Wu, et al. (2016)	(136)	2010					0.5	1	0.75	31	23.25
	Stienstra, Y. et al. (2006)	(137)	2000	Yes	Yes	Yes	Yes	1	0.5	0.75	3	1.5
	Amofah, G. K. et al. (1993)	(138)	1991	Yes				0.95	0.5	0.725	90	64.125
	Thangaraj, H. S., et al. (2000)	(139)	2000		Yes			0.95	0.5	0.725	14	10.15
	Aboagye, S. et al. (2017)	(140)	2015					0.25	1	0.625	3	1.5
	van der Werf, T. S. et al. (1989)	(141)	1987	Yes	Yes	Yes		0.95	0.25	0.6	5	1.875
	Amofah, G. et al. (2002)	(142)	1999					0.5	0.5	0.5	1082	541
	WHO (2001) (CDC)	(19)	2000					0.5	0.5	0.5	572	286
	Duker et al. (2004)	(143)	1999					0.5	0.5	0.5	61	30.5
**Guinea**	WHO (2001) (Sagno)	(19)	2000					0.5	0.5	0.5	221	110.5
**Honduras**	Southern, Paul M. (2016)	(144)	2015	Yes	Yes			0.95	1	0.975	1	0.975
**Japan**	Nakanaga, K. et al. (2013)	(145)	2012	Yes	Yes	Yes	Yes	1	1	1	35	34.625
	Ohtsuka, M. et al. (2014)	(146)	2010	Yes	Yes	Yes		1	1	1	3	3
**Jordan**	Al Ramahi et al. (2017)	(147)	2016	Yes		Yes	Yes	1	1	1	1	1
**Kenya**	Walsh, D. S. et al. (2009)	(148)	2009	Yes			Yes	1	1	1	1	1
**Liberia**	Kollie, K. et al. (2014)	(149)	2012				Yes	1	1	1	21	21
	Monson, MH (1984)	(150)	1981	Yes				1	0.25	0.625	6	3.65
**Malawi**	Komolafe, O. O. (2001)	(151)	2001	Yes				0.95	0.5	0.725	3	2.175
**Mali**	Bessis, D. et al (2015)	(152)	2015				Yes	1	1	1	1	1
	Vignier, N. et al. (2010)	(153)	2014				Yes	1	1	1	1	1
	Ezzedine, K. et al. (2014)	(154)	2007				Yes	1	1	1	1	1
**Mexico**	Coloma, J. N. et al. (2005)	(155)	1997	Yes		Yes	Yes	1	0.5	0.75	2	1.5
	Aguilar et al. (1953)	(156)	1952	Yes		Yes		1	0.25	0.625	1	0.625
**Nigeria**	Ayelo, G. A. et al. (2018)	(157)	2016				Yes	1	1	1	66	66
	Marion, Estelle et al. (2015)	(158)	2013				Yes	1	1	1	64	64
	Ukwaja, K. N., et al. (2016)	(159)	2013	Yes			Yes	1	1	1	36	36
	Chukwuekezie, O. et al. (2007)	(160)	2006				Yes	1	1	1	14	10.5
	Marion, E. et al. (2014 a)	(161)	2013				Yes	1	1	1	1	1
	Ablordey, A. et al. (2015)	(45)	2010				Yes	1	1	1	1	1
	Nigeria MoH (2017)	(162)	2016					0.5	1	0.75	512	384
	Otuh, P. I. et al. (2018)	(163)	2013					0.5	1	0.75	48	36
	Farber et al. (1967)	(164)	1966	Yes				0.95	0.25	0.6	1	0.62
	Gray (1967)	(165)	1966	Yes		Yes		0.95	0.25	0.6	1	0.6
**PNG**	Igo, J. D. et al. (1988)	(166)	1983	Yes		Yes		1	0.25	0.625	46	28.75
	Jacquemart et al. (2002)	(167)	2001					0.5	0.5	0.5	402	201
	WHO (2001) (Joseph)	(19)	2000					0.5	0.5	0.5	12	6
	WHO (2004)	(168)	2003					0.5	0.5	0.5	4	2
	WHO (Joseph) (2000)	(22)	2000					0.5	0.5	0.5	2	1
	Radford, A. J. (2009)	(169)	1966					0.5	0.5	0.5	1	0.5

Country	Reference		Recording	Confirmation method				Scores assigned			Number of cases	
	Main author (year published)	Ref	year	ZN	Culture	Histological	PCR	Diagnosis	Contemporariness	Quality	Included	Adjusted
**Peru**	Guerra, H. et al. (2008)	(170)	2013	Yes		Yes	Yes	1	1	1	8	7.975
	Moyano, Luz M. et al. (2008)	(171)	2008	Yes			Yes	1	1	1	1	1
**Republic of Congo**	Marion, E., et al. (2014 b)	(172)	2012				Yes	1	1	1	12	12
**Sierra Leone**	Murphy, H. E. (2013)	(173)	2003	Yes				0.95	1	0.975	17	16.575
**South Sudan**	South Sudan MoH (2015)	(174)	2005				Yes	1	1	1	33	24.75
	Sindani, I. S. (2006)	(175)	2005	Yes				0.95	1	0.975	30	23.625
	WHO (2003)	(23)	2003					0.5	1	0.75	960	578
**Suriname**	Faber, W. R. et al. (2015)	(176)	2003	Yes	Yes	Yes	Yes	1	0.25	0.625	1	0.625
**Togo**	Beissner, M. et al. (2015)	(177)	2013				Yes	1	1	1	199	199
	Beissner, M. et al. (2013)	(178)	2013				Yes	1	1	1	81	76.75
	Togo MoH (2017)	(179)	2017	Yes			Yes	1	1	1	62	58.75
	Maman, Issaka et al. (2018)	(180)	2015	Yes			Yes	1	1	1	46	34.5
	Beissner, M. et al. (2012)	(181)	2010	Yes			Yes	1	1	1	1	1
	Ablordey, A. et al. (2015)	(45)	2000				Yes	1	0.5	0.75	2	1.5
	Meyers, M. W. et al (1996)	(182)	1994	Yes		Yes	Yes	1	0.5	0.75	2	1.5
**Uganda**	Bradley, D. et al (1970)	(183)	1970	Yes		Yes		1	0.25	0.625	5	2.875
	Clancey, J. et al (1962)	(184)	1962	Yes	Yes	Yes		0.95	0.25	0.6	36	21.6
	Bradley, D. et al (1971)	(185)	1970					0.5	0.25	0.375	220	82.5
	Barker, D. J. (1973 a)	(186)	1972					0.5	0.25	0.375	42	15.75
	Barker, D. J. (1973 b)	(187)	1971					0.5	0.25	0.375	1	0.375

PCR = polymerase chain reaction. ZN = Ziehl Neelsen staining. DRC = Democratic Republic of Congo, PNG = Papua New Guinea, CAR = Central African Republic. [1] Data shared on request.

References for table S.3

1. Kibadi K, et al. New foci of Buruli ulcer, Angola and Democratic Republic of Congo. *Emerg Infect Dis*. 2008
2. Bar W, et al. Mycobacterium ulcerans infection in a child from Angola: diagnosis by direct detection and culture. *Tropical Medicine & International Health*. 1998
3. Tai AYC, Athan E, Friedman ND, et al. Increased Severity and Spread of Mycobacterium ulcerans, Southeastern Australia. *Emerg Infect Dis*. 2018;**24**(1)
4. Boyd SC, et al. Epidemiology, clinical features and diagnosis of Mycobacterium ulcerans in an Australian population. *Med J Aust*. 2012
5. Lavender CJ, et al. Risk of Buruli ulcer and detection of Mycobacterium ulcerans in mosquitoes in southeastern Australia. *PLoS Negl Trop Dis*. 2011
6. Quek TY, et al. Mycobacterium ulcerans infection: factors influencing diagnostic delay. *Med J Aust*. 2007 a
7. Quek TY, et al. Risk factors for Mycobacterium ulcerans infection, southeastern Australia. *Emerg Infect Dis*. 2007 b
8. Steffen CM, Smith, M., McBride, W. J. Mycobacterium ulcerans infection in North Queensland: the 'Daintree ulcer'. *ANZ J Surg*. 2010
9. World Health Organisation. 9e Réunion annuelle de l'Initiative mondiale contre l'ulcère de Buruli de l'OMS; 2006.
10. O'Brien DP, et al. Outcomes for Mycobacterium ulcerans infection with combined surgery and antibiotic therapy: findings from a south-eastern Australian case series. *Medical Journal of Australia*. 2007
11. Johnson PD, et al. Mycobacterium ulcerans in mosquitoes captured during outbreak of Buruli ulcer, southeastern Australia. *Emerg Infect Dis*. 2007
12. Carson C, et al. Potential wildlife sentinels for monitoring the endemic spread of human buruli ulcer in South-East australia. *PLoS Negl Trop Dis*. 2014
13. Francis G, Whitby, M. and Woods, M. Mycobacterium ulcerans infection: a rediscovered focus in the Capricorn Coast region of central Queensland. *Medical Journal of Australia*. 2006
14. van Ravensway J, et al. Climate and landscape factors associated with Buruli ulcer incidence in Victoria, Australia. *PLoS One*. 2012
15. Johnson PD, Veitch MG, Leslie DE, et al. The emergence of Mycobacterium ulcerans infection near Melbourne. *Med J Aust*. 1996
16. Veitch MG, et al. A large localized outbreak of Mycobacterium ulcerans infection on a temperate southern Australian island. *Epidemiol Infect*. 1997
17. Gooding TM, Johnson PD, Smith M, et al. Cytokine profiles of patients infected with Mycobacterium ulcerans and unaffected household contacts. *Infect Immun*. 2002
18. O'Brien DP, et al. Exposure Risk for Infection and Lack of Human-to-Human Transmission of Mycobacterium ulcerans Disease, Australia. *Emerg Infect Dis*. 2017
19. World Health Organisation. 4th WHO advisory group meeting on Buruli ulcer; 2001.
20. Fyfe JAM, et al. A Major Role for Mammals in the Ecology of Mycobacterium ulcerans. *Plos Neglected Tropical Diseases*. 2010
21. Taheri TaF, Sujatha. Buruli Ulcer (Skin Infection with M. Ulcerans): a Case Report and Review of Literature. *Pathology update*. 2009
22. World Health Organisation. 3rd WHO Advisory Group Meeting Report on Buruli Ulcer; 2000.
23. World Health Organisation. Weekly epidemiological record 19. 2003
24. Mitchell PJ, McOrist S, Bilney R. Epidemiology of Mycobacterium ulcerans infection in koalas (Phascolarctos cinereus) on Raymond Island, southeastern Australia. *J Wildl Dis*. 1987;**23**(3):386-90
25. Stinear T, et al. A simple PCR method for rapid genotype analysis of Mycobacterium ulcerans. *J Clin Microbiol*. 2000
26. Amoussouhoui AS, et al. Implementation of a decentralized community-based treatment program to improve the management of Buruli ulcer in the Ouinhi district of Benin, West Africa. *PLoS Negl Trop Dis*. 2018

27. Sopoh GE, et al. Family relationship, water contact and occurrence of Buruli ulcer in Benin. *PLoS Negl Trop Dis*. 2010 a
28. Nackers F, et al. BCG vaccine effectiveness against Buruli ulcer: A case-control study in Benin. *American Journal of Tropical Medicine and Hygiene*. 2006
29. Eddyani M, Vandelannoote K, Meehan CJ, et al. A Genomic Approach to Resolving Relapse versus Reinfection among Four Cases of Buruli Ulcer. *Plos Neglected Tropical Diseases*. 2015;**9**(11)
30. Ruf MT, et al. Secondary Buruli Ulcer Skin Lesions Emerging Several Months after Completion of Chemotherapy: Paradoxical Reaction or Evidence for Immune Protection? *Plos Neglected Tropical Diseases*. 2011
31. Andreoli A, Ruf MT, Sopoh GE, et al. Immunohistochemical Monitoring of Wound Healing in Antibiotic Treated Buruli Ulcer Patients. *Plos Neglected Tropical Diseases*. 2014
32. Barogui YT, Diez G, Anagonou E, et al. Integrated approach in the control and management of skin neglected tropical diseases in Lalo, Benin. *PLoS Negl Trop Dis*. 2018;**12**(6):e0006584
33. Leigheb G, et al. Ultrasonography for the Monitoring of Subcutaneous Damage in Mycobacterium Ulcerans Infection (Buruli Ulcer). *Ultrasound in Medicine and Biology*. 2008
34. Sopoh GE, et al. Severe multifocal form of buruli ulcer after streptomycin and rifampin treatment: comments on possible dissemination mechanisms. *Am J Trop Med Hyg*. 2010 c
35. Wadagni AC, et al. Delayed versus standard assessment for excision surgery in patients with Buruli ulcer in Benin: a randomised controlled trial. *Lancet Infect Dis*. 2018
36. Debacker M, et al. Mycobacterium ulcerans disease (Buruli ulcer) in rural hospital, Southern Benin, 1997–2001. *Emerging infectious diseases*. 2004
37. Johnson RC, Boni G, Barogui Y, et al. Assessment of water, sanitation, and hygiene practices and associated factors in a Buruli ulcer endemic district in Benin (West Africa). *BMC Public Health*. 2015;**15**:801
38. Campbell LP, et al. Spatial Analysis of Anthropogenic Landscape Disturbance and Buruli Ulcer Disease in Benin. *PLoS Negl Trop Dis*. 2015
39. Sopoh G, et al. Distribution of Buruli ulcer in the Ze district of Benin. *Med Trop (Mars)*. 2010 b
40. Barogui Y, et al. Functional Limitations after Surgical or Antibiotic Treatment for Buruli Ulcer in Benin. *American Journal of Tropical Medicine and Hygiene*. 2009
41. Johnson R, Sopoh G, Boko M, et al. Distribution de l'infection à Mycobacterium ulcerans (Ulcère de Buruli) dans la commune de Lalo au Bénin. *Tropical Medicine & International Health*. 2005;**10**(9):863-71
42. Sopoh GE, et al. Buruli ulcer prevalence and altitude, Benin. *Emerg Infect Dis*. 2011
43. Durnez L, et al. Terrestrial small mammals as reservoirs of Mycobacterium ulcerans in benin. *Appl Environ Microbiol*. 2010
44. Williamson HR, et al. Detection of Mycobacterium ulcerans in the environment predicts prevalence of Buruli ulcer in Benin. *PLoS Negl Trop Dis*. 2012
45. Ablordey AS, et al. Whole genome comparisons suggest random distribution of Mycobacterium ulcerans genotypes in a Buruli ulcer endemic region of Ghana. *PLoS Negl Trop Dis*. 2015
46. Abalos FM, Aguiar J, Guédénon A, et al. Mycobacterium ulcerans infection (Buruli ulcer): A case report of the disseminated nonulcerative form. *Ann Diagn Pathol*. 2000
47. Josse R, et al. - Buruli's ulcer, a pathology little known in Benin. Apropos of 227 cases. *Bull Soc Pathol Exot*. 1994
48. Josse R GA, Darie H, Anagonou S, Portaels F, Meyers WM. Etude clinique et epidemiologique de lulcere de buruli chez le jeune au Benin. *Cahiers Sante*. 2002
49. Stoffel V, Barthelme BaC, F. Tropical ecopathology: up hill and down dale Buruli ulcer. *Sante Publique*. 2005
50. dos Santos JL. Mycobacterium ulcerans infection in Brazil. *MJA*. 2007
51. Ouoba K, et al. Cutaneous mycobacterium ulcerans ulcers in Burkina Faso: Six cases report with literature review. [French]. *La Tunisie médicale*. 1998
52. National Yaws, Leishmaniasis, Leprosy and Buruli ulcer Control Programme Cameroon. Buruli ulcer surveillance dataset. Compiled.

53. Christinet V, et al. Impact of human immunodeficiency virus on the severity of buruli ulcer disease: results of a retrospective study in cameroon. *Open Forum Infect Dis*. 2014
54. Landier J, et al. Adequate wound care and use of bed nets as protective factors against Buruli Ulcer: results from a case control study in Cameroon. *PLoS Negl Trop Dis*. 2011
55. Bratschi MW, et al. Geographic distribution, age pattern and sites of lesions in a cohort of Buruli ulcer patients from the Mape Basin of Cameroon. *PLoS Negl Trop Dis*. 2013
56. Marion E, et al. Geographic expansion of Buruli ulcer disease, Cameroon. *Emerg Infect Dis*. 2011
57. Porten K, et al. Prevalence of Buruli ulcer in Akonolinga health district, Cameroon: results of a cross sectional survey. *PLoS Negl Trop Dis*. 2009
58. Bratschi MW, et al. Mycobacterium ulcerans persistence at a village water source of Buruli ulcer patients. *PLoS Negl Trop Dis*. 2014
59. Zambou MV, Takougang, I. and Mbam, L. M. Contribution of community health workers in the control of buruli ulcer in the Ngoantet area, Cameroon. *Tropical Medicine & International Health*. 2011
60. Bolz M, Bratschi MW, Kerber S, et al. Locally Confined Clonal Complexes of Mycobacterium ulcerans in Two Buruli Ulcer Endemic Regions of Cameroon. *PLoS Negl Trop Dis*. 2015;**9**(6):e0003802
61. Awah PK, et al. Developing a Buruli ulcer community of practice in Bankim, Cameroon: A model for Buruli ulcer outreach in Africa. *PLoS Negl Trop Dis*. 2018
62. Wanda F, et al. Clinical features and management of a severe paradoxical reaction associated with combined treatment of Buruli ulcer and HIV co-infection. *Bmc Infectious Diseases*. 2014
63. Andreoli A, Mou F, Minyem JC, et al. Complete Healing of a Laboratory-Confirmed Buruli Ulcer Lesion after Receiving Only Herbal Household Remedies. *PLoS Negl Trop Dis*. 2015;**9**(11):e0004102
64. Landier J, et al. Spatio-temporal patterns and landscape-associated risk of Buruli ulcer in Akonolinga, Cameroon. *PLoS Negl Trop Dis*. 2014
65. Um Boock A. Enquete Preliminaire Nationale sur la Situation de l'Ulcere de Buruli au Cameroun (Phd Thesis) 2004.
66. Zogo B, et al. A Field Study in Benin to Investigate the Role of Mosquitoes and Other Flying Insects in the Ecology of Mycobacterium ulcerans. *PLoS Negl Trop Dis*. 2015
67. Noeske J, et al. Buruli ulcer disease in Cameroon rediscovered. *Am J Trop Med Hyg*. 2004
68. Ebong SM, et al. Survey of water bugs in bankim, a new buruli ulcer endemic area in cameroon. *J Trop Med*. 2012
69. Minime-Lingoupou F, et al. Buruli ulcer, Central African Republic. *Emerg Infect Dis*. 2010
70. Faber WR, et al. First reported case of Mycobacterium ulcerans infection in a patient from China. *Trans R Soc Trop Med Hyg*. 2000
71. N'Krumah R TAS, Kone B, Cisse G, et al. Characteristics and epidemiological profile of Buruli ulcer in the district of Tiassale, south Cote d'Ivoire. *Acta Trop*. 2017;**175**:138-44
72. N'Krumah R T, et al. Socio-Environmental Factors Associated with the Risk of Contracting Buruli Ulcer in Tiassale, South Cote d'Ivoire: A Case-Control Study. *PLoS Negl Trop Dis*. 2016
73. Coulibaly-N'Golo GMD, et al. Multilocus VNTR analysis of Mycobacterium ulcerans strains isolated in Cote d'Ivoire. *Journal of Infection in Developing Countries*. 2011
74. Kouame K, et al. Buruli ulcer involving the head: outcomes and therapeutic aspects in 8 cases observed at the University Hospital Center in Abidjan, Cote d'Ivoire. *Med Trop (Mars)*. 2008
75. Sangare A. L'Atteinte Palpabrale Bilaterale : une Localisation Rare de l'Ulcere de Buruli. *Rev Int Sc Méd*. 2007
76. Ahoua et al. Risk factors for Buruli ulcer in Côte d'Ivoire: Results of a case-control study, August 2001. *Afr J Biotechnol*. 2009
77. Ecra E, et al. - Detection and treatment of early forms of Mycobacterium ulcerans infection in Ivory Coast. *0*. 2005
78. Konan KL, et al. Detection of the IS2404 insertion sequence and ketoreductase produced by Mycobacterium ulcerans in the aquatic Heteroptera in the health districts of Dabou and Tiassale in Cote d'Ivoire. *Med Sante Trop*. 2015

79. Boni CC, et al. Comparative study of the flora in the fields of water courses in the hypo and hyper endemic zones of Buruli ulcer in Cote d'Ivoire. [French]. *Int J Biol Chem Sci* 2017
80. Darie H, Le Guyadec T, Touze JE. Epidemiological and clinical aspects of Buruli ulcer in Ivory Coast. 124 recent cases. *Bull Soc Pathol Exot*. 1993;**86**(4):272-6
81. Richard-Kadio. Les Ulceres Cutanes a Mycobacteries Atypiques et leur Traitement par la Chirurgie Plastique. *Trans R Soc Trop Med Hyg*. 1990
82. Kanga JM. Epidemiology of Buruli ulcer in Cote d'Ivoire: results of a national survey. *Bulletin De La Societe De Pathologie Exotique*. 2001
83. Ecra EJ, et al. Buruli ulcer: report of two thoraco-abdominal cases associated with pleurisy. [French]. *Médecine d'Afrique Noire*. 2001
84. Espey DK, Djomand G, Diomande I, et al. A pilot study of treatment of Buruli ulcer with rifampin and dapsone. *Int J Infect Dis*. 2002;**6**(1):60-5
85. Marston B, Diallo MO, Horsburgh CR, Diomande I. Emergence of Buruli ulcer Disease in the Daloa region of Côte d'Ivoire. *Am J Trop Med Hyg*. 1995
86. Mavinga Phanzu D, et al. Burden of Mycobacterium ulcerans disease (Buruli ulcer) and the underreporting ratio in the territory of Songololo, Democratic Republic of Congo. *PLoS Negl Trop Dis*. 2013
87. Mavinga Phanzu D, Suykerbuyk P, Imposo DB, et al. Effect of a control project on clinical profiles and outcomes in buruli ulcer: a before/after study in Bas-Congo, Democratic Republic of Congo. *PLoS neglected tropical diseases*. 2011 a;**5**(12):e1402
88. Kibadi K, et al. Response to Treatment in a Prospective Cohort of Patients with Large Ulcerated Lesions Suspected to Be Buruli Ulcer (Mycobacterium ulcerans Disease). *Plos Neglected Tropical Diseases*. 2010
89. Suykerbuyk P, et al. Persistence of Mycobacterium ulcerans disease (Buruli Ulcer) in the historical focus of Kasongo Territory, the Democratic Republic of Congo. *Am J Trop Med Hyg*. 2009
90. Mavinga Phanzu D, et al. Mycobacterium ulcerans disease (Buruli ulcer) in a rural hospital in Bas-Congo, Democratic Republic of Congo, 2002-2004. *Am J Trop Med Hyg*. 2006
91. Kibadi K, Boelaert M, Kayinua M, et al. Therapeutic itineraries of patients with ulcerated forms of Mycobacterium ulcerans (Buruli ulcer) disease in a rural health zone in the Democratic Republic of Congo. *Trop Med Int Health*. 2009 a;**14**(9):1110-6
92. Mavinga Phanzu D, Mahema RL, Suykerbuyk P, et al. Mycobacterium ulcerans infection (Buruli ulcer) on the face: a comparative analysis of 13 clinically suspected cases from the Democratic Republic of Congo. *The American journal of tropical medicine and hygiene*. 2011 b;**85**(6):1100-5
93. Kibadi K. A rare large cutaneous ulcer from the rural area, Democratic Republic of Congo. What is the diagnosis: Buruli ulcer? *Rural Remote Health*. 2009 b;**9**(4)
94. Mavinga Phanzu D, et al. Short report: Edematous Mycobacterium ulcerans infection (Buruli ulcer) on the face: A case report. *American Journal of Tropical Medicine and Hygiene*. 2007
95. Delaporte E, Alfandari S, Piette F. Mycobacterium ulcerans associated with infection due to the human immunodeficiency virus. *Clinical infectious diseases : an official publication of the Infectious Diseases Society of America*. 1994;**18**(5):839
96. Meyers WM, Connor DH, McCullough B, et al. Distribution of Mycobacterium ulcerans infections in Zaire, including the report of new foci. *Ann Soc Belg Med Trop*. 1974;**54**(3):147-57
97. Smith JH. Epidemiologic Observations on Cases of Buruli Ulcer Seen in a Hospital in Lower Congo. *American Journal of Tropical Medicine and Hygiene*. 1970;**19**(4):657-&
98. Andersen FO. Mycobacterial Skin Ulcers--Clinical Experience. *The Central African journal of medicine*. 1965;**11**:131-5
99. Meyers WM, Connor DH. Mycobacterium ulcerans infections in leprosy patients. *Lepr Rev*. 1975;**46**(1):21-7
100. Guerden A. Discussion des communications. *Ann Soc Belge Med Trop*. 1962
101. Pattyn SR. Bacteriology and human and experimental pathology of ulcers caused by Mycobacterium ulcerans. *Sante Publique*. 1965
102. Hennebert P, Gatti F, Vandepitte J, et al. Deux cas d'ulcère nécrotique à BAR observés à Léopoldville. *Ann Soc belge Méd trop*. 1962;**4**:549-54

103. Gordon D, Zelalem M, Schutze GE, et al. Suspected Buruli ulcer of the face: a case report from Ethiopia. *The Pediatric infectious disease journal*. 2014;**33**(3):323-5
104. Haileamlak A, Girma, B. Buruli Ulcer: The First Reported Case from Ethiopia. *Ethiopian Journal of Health Sciences*. 2009
105. Reynaud Y, et al. Heterogeneity among Mycobacterium ulcerans from French Guiana revealed by multilocus variable number tandem repeat analysis (MLVA). *Plos One*. 2015
106. Douine M, Gozlan R, Nacher M, et al. Mycobacterium ulcerans infection (Buruli ulcer) in French Guiana, South America, 1969-2013: an epidemiological study. *Lancet Planet Health*. 2017;**1**(2):e65-e73
107. Menard A, Couppie P, Sainte-Marie D, et al. Diagnosis of Mycobacterium ulcerans infection by PCR: report of 3 cases observed in French Guiana. *Bull Soc Pathol Exot*. 2003;**96**(5):403-5
108. Bayonne Manou LS, et al. [Mycobacterium ulcerans disease (Buruli ulcer) in Gabon: 2005-2011]. *Med Sante Trop*. 2013
109. Yeboah-Manu D, Aboagye SY, Asare P, et al. Laboratory confirmation of Buruli ulcer cases in Ghana, 2008-2016. *PLoS Negl Trop Dis*. 2018;**12**(6):e0006560
110. Richard Phillips' Buruli ulcer group, Kumasi Centre for Collaborative Research and Komfo Anokye Teaching Hospital. Buruli ulcer patient dataset. Compiled by: Frimpong M. Version 1. 2017.
111. National Buruli Ulcer Control Program, Ghana. Buruli ulcer surveillance dataset. Compiled 2014.
112. Amissah NA, et al. Investigating the role of free-living amoebae as a reservoir for Mycobacterium ulcerans. *PLoS Negl Trop Dis*. 2014
113. Sarfo FS, et al. Clinical Efficacy of Combination of Rifampin and Streptomycin for Treatment of Mycobacterium ulcerans Disease. *Antimicrobial Agents and Chemotherapy*. 2010
114. Bibert S, Bratschi MW, Aboagye SY, et al. Susceptibility to Mycobacterium ulcerans Disease (Buruli ulcer) Is Associated with IFNG and iNOS Gene Polymorphisms. *Front Microbiol*. 2017;**8**
115. Adu EJ. Management of complications of Mycobacterium ulcerans disease: A three-year review. *Int J Mycobacteriol*. 2013
116. Roltgen K, et al. Single nucleotide polymorphism typing of Mycobacterium ulcerans reveals focal transmission of buruli ulcer in a highly endemic region of Ghana. *PLoS Negl Trop Dis*. 2010
117. Phillips RO, et al. Sensitivity of PCR Targeting Mycobacterium ulcerans by Use of Fine-Needle Aspirates for Diagnosis of Buruli Ulcer. *Journal of Clinical Microbiology*. 2009
118. Aboagye SY, et al. Seasonal pattern of Mycobacterium ulcerans, the causative agent of Buruli ulcer, in the environment in Ghana. *Microb Ecol* 2017 a
119. Sarfo FS, et al. Mycobacterium ulcerans DNA Not Detected in Faecal Samples from Buruli Ulcer Patients: Results of a Pilot Study. *Plos One*. 2011
120. Amissah NA, Glasner C, Ablordey A, et al. Genetic diversity of Staphylococcus aureus in Buruli ulcer. *PLoS Negl Trop Dis*. 2015;**9**(2):e0003421
121. Yeboah-Manu D, Roltgen K, Opare W, et al. Sero-Epidemiology as a Tool to Screen Populations for Exposure to Mycobacterium ulcerans. *Plos Neglected Tropical Diseases*. 2012;**6**(1)
122. Narh CA, et al. Source tracking Mycobacterium ulcerans infections in the Ashanti region, Ghana. *PLoS Negl Trop Dis*. 2015
123. Ampah KA, et al. Burden and Historical Trend of Buruli Ulcer Prevalence in Selected Communities along the Offin River of Ghana. *PLoS Negl Trop Dis*. 2016
124. Iddrisah FN, et al. Exposure of Small-Scale Gold Miners in Prestea to Mercury, Ghana, 2012. *Pan Afr Med J*. 2016
125. Sarfo FS, Thompson W, Phillips RO, et al. A severe case of Buruli ulcer disease with pleural effusions. *PLoS neglected tropical diseases*. 2014;**8**(6):e2868
126. Hospers IC, et al. Distribution of Buruli ulcer lesions over body surface area in a large case series in Ghana: uncovering clues for mode of transmission. *Trans R Soc Trop Med Hyg*. 2005
127. Wu J, et al. Buruli Ulcer Disease and Its Association with Land Cover in Southwestern Ghana. *PLoS Negl Trop Dis*. 2015
128. Ackumey MM, Kwakye-Maclean C, Ampadu EO, et al. Health Services for Buruli Ulcer Control: Lessons from a Field Study in Ghana. *PLoS neglected tropical diseases*. 2011;**5**(6)
129. Kenu E, et al. Application of geographical information system (GIS) technology in the control of Buruli ulcer in Ghana. *BMC Public Health*. 2014

130. Raghunathan PL, et al. Risk factors for Buruli ulcer disease (Mycobacterium ulcerans Infection): results from a case-control study in Ghana. *Clin Infect Dis*. 2005
131. Hamzat TK, Boakye-Afram B. Health-related quality of life among persons living with buruli ulcer in amasaman community, ga west district accra, ghana. *International journal of health sciences*. 2011;**5**(1):29-38
132. Kotey NK, Ampadu X. Antibiotic treatment outcomes of Buruli ulcer in Akwapem South and Suhum-Kraboa-Coaltar districts of Eastern region, Ghana. *Trop Med Int Health*. 2011;**16**:195-
133. Osei-Sarpong F. Analysis of Buruli Ulcer Prevalence in Amansie West District: A Geostatistical Approach. *Austin Biom and Biostat* 2015
134. Tschakert P, et al. Situated knowledge of pathogenic landscapes in Ghana: Understanding the emergence of Buruli ulcer through qualitative analysis. *Soc Sci Med*. 2016
135. Williamson HR, et al. Distribution of Mycobacterium ulcerans in buruli ulcer endemic and non-endemic aquatic sites in Ghana. *PLoS Negl Trop Dis*. 2008
136. Wu JY, Smithwick EAH. Landscape Fragmentation as a Risk Factor for Buruli Ulcer Disease in Ghana. *American Journal of Tropical Medicine and Hygiene*. 2016;**95**(1):63-9
137. Stienstra Y, et al. Susceptibility to Buruli ulcer is associated with the SLC11A1 (NRAMP1) D543N polymorphism. *Genes Immun*. 2006
138. Amofah GK, Sagoe-Moses C, Adjei-Acquah C, et al. Epidemiology of Buruli ulcer in Amansie West district, Ghana. *Transactions of the Royal Society of Tropical Medicine and Hygiene*. 1993;**87**(6):644-5
139. Thangaraj HS, et al. In vitro activity of ciprofloxacin, sparfloxacin, ofloxacin, amikacin and rifampicin against Ghanaian isolates of Mycobacterium ulcerans. *Journal of Antimicrobial Chemotherapy*. 2000
140. Aboagye SY, Asare P, Otchere ID, et al. Environmental and Behavioral Drivers of Buruli Ulcer Disease in Selected Communities Along the Densu River Basin of Ghana: A Case-Control Study. *Am J Trop Med Hyg*. 2017 b;**96**(5):1076-83
141. van der Werf TS, van der Graaf WT, Groothuis DG, et al. Mycobacterium ulcerans infection in Ashanti region, Ghana. *Transactions of the Royal Society of Tropical Medicine and Hygiene*. 1989;**83**(3):410-3
142. Amofah G, Bonsu F, Tetteh C, et al. Buruli ulcer in Ghana: results of a national case search. *Emerging infectious diseases*. 2002;**8**(2):167-70
143. Duker AA, Carranza EJ, Hale M. Spatial dependency of Buruli ulcer prevalence on arsenic-enriched domains in Amansie West District, Ghana: implications for arsenic mediation in Mycobacterium ulcerans infection. *International journal of health geographics*. 2004;**3**(1):19
144. Southern PM. Probable Buruli Ulcer Disease in Honduras. *Open Forum Infect Dis*. 2016;**3**(2):ofv189
145. Nakanaga K, Yotsu RR, Hoshino Y, et al. Buruli ulcer and mycolactone-producing mycobacteria. *Jpn J Infect Dis*. 2013;**66**(2):83-8
146. Ohtsuka M, et al. Buruli ulcer caused by Mycobacterium ulcerans subsp shinshuense: a rare case of familial concurrent occurrence and detection of insertion sequence 2404 in Japan. *JAMA Dermatol*. 2014
147. Al Ramahi JW, Annab H, Al Karmi M, et al. Chronic cutaneous mycobacterial ulcers due to Mycobacterium ulcerans (Buruli ulcer): the first indigenous case report from Jordan and a literature review. *International journal of infectious diseases : IJID : official publication of the International Society for Infectious Diseases*. 2017;**58**:77-81
148. Walsh DS, et al. Short report: Clinical and molecular evidence for a case of Buruli ulcer (Mycobacterium ulcerans infection) in Kenya. *Am J Trop Med Hyg*. 2009
149. Kollie K, et al. Buruli ulcer in Liberia, 2012. *Emerg Infect Dis*. 2014
150. Monson M, Gibson D, Connor D, et al. Mycobacterium ulcerans in Liberia: a clinicopathologic study of 6 patients with Buruli ulcer. *Acta tropica*. 1984
151. Komolafe OO. Buruli ulcer in Malawi - a first report. *Malawi Med J*. 2001;**13**(3):37-8
152. Bessis D, Kempf M, Marsollier L. Mycobacterium ulcerans disease (Buruli ulcer) in Mali: A new potential African endemic country. *Acta Derm Venereol*. 2015;**95**(4):489-90
153. Vignier N, et al. Buruli ulcer acquired in Mali: a rare and atypical situation. *Travel Med Infect Dis*. 2014

154. Ezzedine K, Pistone T, Guir V, et al. Painful Buruli Ulcer in a Malian Visitor to France. *Acta Derm-Venereol*. 2010;**90**(4):424-
155. Coloma JN, Navarrete-Franco G, Iribe P, et al. Ulcerative cutaneous mycobacteriosis due to Mycobacterium ulcerans: report of two Mexican cases. *International journal of leprosy and other mycobacterial diseases : official organ of the International Leprosy Association*. 2005;**73**(1):5-12
156. Aguilar PL, Iturribarria FM, Middlebrook G. - A case of human infection by Mycobacterium ulcerans in the western hemisphere; preliminary note. 1953;- **21**(- 4:1):- 476
157. Ayelo GA, et al. Report of a series of 82 cases of Buruli ulcer from Nigeria treated in Benin, from 2006 to 2016. *PLoS Negl Trop Dis*. 2018
158. Marion E, et al. Buruli Ulcer in South Western Nigeria: A Retrospective Cohort Study of Patients Treated in Benin. *PLoS Neglected Tropical Diseases*. 2015
159. Ukwaja KN, et al. Buruli ulcer in Nigeria: results of a pilot case study in three rural districts. *Infect Dis Poverty*. 2016
160. Chukwuekezie O, et al. Buruli ulcer, Nigeria. *Emerg Infect Dis*. 2007
161. Marion E, Chauty A, Yeramian E, et al. A case of guilt by association: Water bug bite incriminated in M. ulcerans infection. *Int J Mycobacteriol*. 2014 a;**3**(2):158-61
162. The National Tuberculosis, Leprosy and Buruli Ulcer Control Programme, Nigeria. Buruli ulcer surveillance dataset. Compiled 2018.
163. Otuh PI, Soyinka FO, Ogunro BN, et al. Perception and incidence of Buruli ulcer in Ogun State, South West Nigeria: intensive epidemiological survey and public health intervention recommended. *Pan Afr Med J*. 2018;**29**:166
164. Farber ER, and Tsang, A. Mycobacterial ('Buruli') ulcer in a Peace Corps worker. *Arch Surg*. 1967
165. Gray. Mycobacterial Skin Ulcers in Nigeria. *Int J Lepr*. 1967
166. Igo JD, Murthy DP. Mycobacterium ulcerans infections in Papua New Guinea: correlation of clinical, histological, and microbiologic features. *Am J Trop Med Hyg*. 1988;**38**(2):391-2
167. Jacquemart YaJ, R. Papouasie Nouvelle-Guinée. *Médecine tropicale*. 2002
168. World Health Organisation. Weekly epidemiological record 2004
169. Radford AJ. The surgical management of lesions of ulcerans infections due to Mycobacterium ulcerans, revisited. *Transactions of the Royal Society of Tropical Medicine and Hygiene*. 2009
170. Guerra H, et al. Mycobacterium ulcerans disease, Peru. *Emerg Infect Dis*. 2008
171. Moyano LM, Chero JC, Gonzalvez GE, et al. Buruli ulcer. *Am J Trop Med Hyg*. 2008;**79**(1):3
172. Marion E, et al. Buruli ulcer disease in Republic of the Congo. *Emerg Infect Dis*. 2014 b
173. Murphy HE. Buruli Ulcer In Sierra Leone: An Unique Undergraduate Elective Project Prize-winning Pathological Society Award for Best Elective Report 2011. *Journal of Pathology*. 2013
174. South Sudan MoH. South Sudan Master Plan for Neglected Tropical Diseases. 2015
175. Sindani IS. Report II of the Uganda Buruli Group; 2006.
176. Faber WR, de Jong B, de Vries HJ, et al. Buruli ulcer in traveler from Suriname, South America, to the Netherlands. *Emerg Infect Dis*. 2015;**21**(3):497-9
177. Beissner M, et al. Treatment Outcome of Patients with Buruli Ulcer Disease in Togo. *PLoS Negl Trop Dis*. 2015
178. Beissner M, et al. Implementation of a national reference laboratory for Buruli ulcer disease in Togo. *PLoS Negl Trop Dis*. 2013
179. National Reference Laboratory for Buruli ulcer disease in Togo. Buruli ulcer surveillance dataset.
180. Maman I, Tchacondo T, Kere AB, et al. Risk factors for Mycobacterium ulcerans infection (Buruli Ulcer) in Togo horizontal line a case-control study in Zio and Yoto districts of the maritime region. *BMC Infect Dis*. 2018;**18**(1):48
181. Beissner M, et al. Spontaneous Clearance of a Secondary Buruli Ulcer Lesion Emerging Ten Months aftera Completion of Chemotherapy-A Case Report from Togo. *Plos Neglected Tropical Diseases*. 2012
182. Meyers MW et al. Mycobacterium ulcerans infection (Buruli ulcer): first reported patients in Togo. *Br J Dermatol*. 1996
183. Bradley DJ et al. Clinical Features and Treatment of Pre-Ulcerative Buruli Lesions: REPORT II OF THE UGANDA BURULI GROUP. *Br Med J*. 1970

184. Clancey J, Dodge RaL, H. F. Study of a mycobacterium causing skin ulceration in Uganda. *Ann Soc Belg Med Trop (1920)*. 1962
185. Bradley DJ et al. Epidemiology of Mycobacterium Ulcerans Infection (Buruli Ulcer) at Kinyara, Uganda. *Trans R Soc Trop Med Hyg*. 1971
186. Barker DJ. Epidemiology of Mycobacterium ulcerans infection. *Trans R Soc Trop Med Hyg*. 1973 a;**67**(1):43-50
187. Barker DJPaC, J. W. Mycobacterium Ulcerans Infection among Tsetse Control Workers in Uganda. *International Journal of Epidemiology*. 1973 b

Table S.4: Data extracted from selected studies used for evidence consensus framework- detection of *M. ulcerans* in environmental and animal samples

Country	Main author (year published)	Ref	Year	Sample type	PCR targets				qPCR confirmed	VNTR/MIRU results		other results	MU confirmed	N. positive samples
					ER	KR	IS2404	IS2606		MU	other MPM			
**Australia**	Carson et al. (2014)	(1)	2013	terr. vert. fae.		+ve	+ve	+ve	yes				yes	23
	Elsner et al. (2008)	(2)	2006	terr. vert. clin.			+ve					Clinical diagnosis	yes	1
	Fyfe et al. (2010)	(2)	2009	terr. vert. fae.		+ve	+ve	+ve	yes			ZN +	yes	70
	Fyfe et al. (2010)	(2)	2009	terr. vert. fae.		+ve	+ve	+ve	yes				yes	82
	Johnson et al. (2007)	(3)	2007	terr. invert.			+ve				+ve		no	768
	McOrist et al. (1985)	(4)	1985	terr. vert. clin.								ZN+ and culture+	yes	2
	Mitchell et al. (1984)	(5)	1980	terr. vert. clin.								histopathology +, ZN+ and culture+	yes	7
	Mitchell et al. (1987)	(6)	1985	terr. vert. clin.								Clinical diagnosis	no	8
	O'Brien et al. (2014)	(7)	1998	terr. vert. clin.			+ve					Clinical diagnosis	yes	1
	O'Brien et al. (2014)	(7)	2000	terr. vert. clin.			+ve					Clinical diagnosis	yes	2
	O'Brien et al. (2014)	(7)	2001	terr. vert. clin.			+ve					Clinical diagnosis	yes	2
	O'Brien et al. (2014)	(7)	2002	terr. vert. clin.			+ve					Clinical diagnosis	yes	2
	O'Brien et al. (2014)	(7)	2003	terr. vert. clin.			+ve					Clinical diagnosis	yes	2
	O'Brien et al. (2014)	(7)	2005	terr. vert. clin.			+ve					Clinical diagnosis	yes	1
	O'Brien et al. (2014)	(7)	2007	terr. vert. clin.			+ve					Clinical diagnosis	yes	1
	O'Brien et al. (2014)	(7)	2008	terr. vert. clin.			+ve					Clinical diagnosis	yes	6
	O'Brien et al. (2014)	(7)	2009	terr. vert. clin.			+ve					Clinical diagnosis	yes	8
	O'Brien et al. (2014)	(7)	2010	terr. vert. clin.			+ve					Clinical diagnosis	yes	10
	O'Brien et al. (2014)	(7)	2011	terr. vert. clin.			+ve					Clinical diagnosis	yes	1
	O'Brien et al. (2014)	(7)	2012	terr. vert. clin.			+ve					Clinical diagnosis	yes	4
	Röltgen et al. (2017)	(8)	2013	terr. invert.		+ve	+ve	+ve	yes				yes	1
	Röltgen et al. (2017)	(8)	2013	terr. vert. fae.		+ve	+ve	+ve	yes				yes	2
	Röltgen et al. (2017)	(8)	2013	terr. vert. fae.			+ve						yes	1
	Ross et al. (1997)	(9)	1995	aq non-animal			+ve						no	6
	WHO (2001) (Johnson)	(10)	2001	terr. vert. clin.			+ve						yes	3
**Benin**	Djouaka et al. (2017)	(11)	2016	aq. animal		-ve	+ve	-ve	no				no	16
	Djouaka et al. (2017)	(11)	2016	aq. animal		-ve	+ve	+ve	no				no	34
	Djouaka et al. (2017)	(11)	2016	terr. invert.		-ve	+ve	-ve	no				no	240
	Djouaka et al. (2017)	(11)	2016	terr. invert.		+ve	+ve	-ve	no				no	120
	Djouaka et al. (2018)	(12)	2018	terr. vert clin		-ve	+ve	-ve	yes				yes	3

Country	Main author (year published)	Ref	Year	Sample type	PCR targets				qPCR confirmed	VNTR/MIRU results		other results	MU confirmed	N. positive samples
					ER	KR	IS2404	IS2606		MU	other MPM			
**Benin**	Djouaka et al. (2018)	(12)	2018	terr. vert. clin		-ve	+ve	+ve	yes				yes	3
	Djouaka et al. (2018)	(12)	2018	terr. vert. clin		+ve	+ve	+ve	yes	+ve			yes	2
	Eddyani et al. (2004)*	(13)	2001	aq. animal			+ve						no	1
	Williamson et al. (2012)	(14)	2012	aq. non-animal	-ve		+ve						no	39
	Williamson et al. (2012)	(14)	2012	aq. non-animal	+ve		+ve		no				no	8
	Williamson et al. (2012)	(14)	2012	aq. non-animal	+ve		+ve		yes				yes	12
	Williamson et al. (2012)	(14)	2012	aq. non-animal	+ve		+ve						no	46
	Zogo et al. (2015)	(15)	2013	aq. animal	+ve		+ve		yes				yes	31
	Zogo et al. (2015)	(15)	2013	aq. non-animal			+ve		yes				yes	1
**Cameroon**	Bratschi et al. (2014)	(16)	2011	aq. non-animal		+ve	+ve	+ve	yes				yes	3
	Djouaka et al. (2018)	(12)	2018	terr. vert. clin		-ve	+ve	+ve	yes				yes	3
	Djouaka et al. (2018)	(12)	2018	terr. vert. clin			+ve		yes				yes	5
	Garchitorena et al. (2014)	(17)	2013	aq. animal		+ve	+ve		yes				yes	3084
	Marion et al. (2010)	(18)	2008	aq. animal		+ve	+ve		yes		+ve		yes	1
**Côte d'Ivoire**	Konan et al. (2015)	(19)	2008	aq. animal	+ve		+ve		yes				no	26
	Tano et al. (2017)	(20)	2017	aq. non-animal	+ve		+ve			-ve			no	15
**French Guiana**	Morris et al. (2014)	(21)	2014	aq. non-animal		-ve	+ve						no	6
	Morris et al. (2014)	(21)	2014	aq. non-animal		+ve	+ve		yes				yes	3
**Ghana**	Aboagye et al. (2017)	(22)	2014	aq. non-animal		+ve	+ve	+ve	yes				yes	237
	Amissah et al. (2014)	(23)	2009	aq. non-animal			+ve						no	8
	Benbow et al. (2014)	(24)	2007	aq. non-animal	+ve					+ve			yes	34
	Benbow et al. (2014)	(24)	2007	aq. non-animal	+ve						+ve		no	10
	Benbow et al. (2014)	(24)	2007	aq. non-animal	+ve								no	37
	Eddyani et al. (2004)*	(13)	2001	aq. animal			-ve						no	1
	Eddyani et al. (2004)*	(13)	2001	aq. animal			+ve						no	3
	Narh et al. (2015)	(25)	2015	aq. non-animal	+ve		+ve			+ve			no	9
	Narh et al. (2015)	(25)	2015	terr. vert. clin.	+ve		+ve						no	1

Country	Main author (year published)	Ref	Year	Sample type	PCR targets				qPCR confirmed	VNTR/MIRU results		other results	MU confirmed	N. positive samples
					ER	KR	IS2404	IS2606		MU	other MPM			
**Ghana**	Tobias et al. (2016)	(26)	2013	terr. vert. fae.		-ve	-ve						no	7
	Tobias et al. (2016)	(26)	2013	terr. vert. fae.			-ve						no	14
	Tobias et al. (2016)	(26)	2013	terr. vert. fae.			+ve						no	4
	Vandelannoote et al. (2010)	(27)	2010	aq. non-animal		+ve	+ve		yes				yes	1
	Vandelannoote et al. (2010)	(27)	2010	aq. non-animal			+ve		yes				yes	2
	Williamson et al. (2008)	(28)	2006	aq. non-animal	-ve								no	7
	Williamson et al. (2008)	(28)	2006	aq. non-animal	+ve					-ve	-ve		no	1
	Williamson et al. (2008)	(28)	2006	aq. non-animal	+ve					-ve			yes	5
	Williamson et al. (2008)	(28)	2006	aq. non-animal	+ve					-ve	+ve		yes	2
	Williamson et al. (2008)	(28)	2006	aq. non-animal	+ve					+ve	-ve		yes	7
	Williamson et al. (2008)	(28)	2006	aq. non-animal	+ve					+ve	+ve		yes	4
	Willson et al. (2013)	(29)	2008	aq. animal						+ve			yes	1
	Willson et al. (2013)	(29)	2008	aq. non-animal	+ve					+ve			yes	2
**Japan**	Ohtsuka et al. (2014)	(30)	2010	aq. animal			+ve						no	1
**Togo**	Maman et al. (2018)	(31)	2015	aq. non-animal	na	-ve	+ve	-ve	-ve	na			no	4
	Maman et al. (2018)	(31)	2015	aq. non-animal	na	-ve	+ve	+ve	-ve	na			no	4
	Maman et al. (2018)	(31)	2015	aq. non-animal	na	+ve	+ve	+ve	yes	na			yes	4
**USA**	Hennigan et al. (2013)	(32)	2011	aq. non-animal			+ve						no	66

Terr. = terrestrial; Aq. = aquatic; vert. = vertebrate; invert. = invertebrate; fae. = faeces; clin. = clinical.

References for table S.4

1. Carson C, Lavender CJ, Handasyde KA, O'Brien CR, Hewitt N, Johnson PD, et al. Potential wildlife sentinels for monitoring the endemic spread of human buruli ulcer in South-East australia. PLoS neglected tropical diseases. 2014;8(1):e2668.
2. Fyfe JA, Lavender CJ, Handasyde KA, Legione AR, O'Brien CR, Stinear TP, et al. A major role for mammals in the ecology of Mycobacterium ulcerans. PLoS neglected tropical diseases. 2010;4(8):e791.
3. Johnson PD, Azuolas J, Lavender CJ, Wishart E, Stinear TP, Hayman JA, et al. Mycobacterium ulcerans in mosquitoes captured during outbreak of Buruli ulcer, southeastern Australia. Emerging infectious diseases. 2007;13(11):1653-60.
4. McOrist S, Jerrett IV, Anderson M, Hayman J. Cutaneous and respiratory tract infection with Mycobacterium ulcerans in two koalas (Phascolarctos cinereus). J Wildl Dis. 1985;21(2):171-3.
5. Mitchell PJ, Jerrett IV, Slee KJ. Skin ulcers caused by Mycobacterium ulcerans in koalas near Bairnsdale, Australia. Pathology. 1984;16(3):256-60.
6. Mitchell PJ, McOrist S, Bilney R. Epidemiology of Mycobacterium ulcerans infection in koalas (Phascolarctos cinereus) on Raymond Island, southeastern Australia. Journal of Wildlife Diseases. 1987;23(3):386-90.
7. O'Brien CR, Handasyde KA, Hibble J, Lavender CJ, Legione AR, McCowan C, et al. Clinical, microbiological and pathological findings of Mycobacterium ulcerans infection in three Australian Possum species. PLoS neglected tropical diseases. 2014;8(1):e2666.
8. Roltgen K, Pluschke G, Johnson PDR, Fyfe J. Mycobacterium ulcerans DNA in Bandicoot Excreta in Buruli Ulcer-Endemic Area, Northern Queensland, Australia. Emerging infectious diseases. 2017;23(12):2042-5.
9. Ross BC, Johnson PD, Oppedisano F, Marino L, Sievers A, Stinear T, et al. Detection of Mycobacterium ulcerans in environmental samples during an outbreak of ulcerative disease. Applied and environmental microbiology. 1997;63(10):4135-8.
10. World Health Organisation, editor 4th WHO Advisory Group Meeting on Buruli Ulcer2001.
11. Djouaka R, Zeukeng F, Daiga Bigoga J, N'Golo Coulibaly D, Tchigossou G, Akoton R, et al. Evidences of the Low Implication of Mosquitoes in the Transmission of Mycobacterium ulcerans, the Causative Agent of Buruli Ulcer. The Canadian journal of infectious diseases & medical microbiology = Journal canadien des maladies infectieuses et de la microbiologie medicale. 2017;2017:1324310.
12. Djouaka R, Zeukeng F, Bigoga JD, Kakou-Ngazoa SE, Akoton R, Tchigossou G, et al. Domestic animals infected with Mycobacterium ulcerans-Implications for transmission to humans. PLoS neglected tropical diseases. 2018;12(7):e0006572.
13. Eddyani M, Ofori-Adjei D, Teugels G, De Weirdt D, Boakye D, Meyers WM, et al. Potential role for fish in transmission of Mycobactetium ulcerans disease (Buruli ulcer): an environmental study. Applied and environmental microbiology. 2004;70(9):5679-81.
14. Williamson HR, Benbow ME, Campbell LP, Johnson CR, Sopoh G, Barogui Y, et al. Detection of Mycobacterium ulcerans in the environment predicts prevalence of Buruli ulcer in Benin. PLoS neglected tropical diseases. 2012;6(1):e1506.
15. Zogo B, Djenontin A, Carolan K, Babonneau J, Guegan JF, Eyangoh S, et al. A Field Study in Benin to Investigate the Role of Mosquitoes and Other Flying Insects in the Ecology of Mycobacterium ulcerans. PLoS neglected tropical diseases. 2015;9(7):e0003941.
16. Bratschi MW, Bolz M, Grize L, Kerber S, Minyem JC, Um Boock A, et al. Primary cultivation: factors affecting contamination and Mycobacterium ulcerans growth after long turnover time of clinical specimens. BMC infectious diseases. 2014;14:636.
17. Garchitorena A, Roche B, Kamgang R, Ossomba J, Babonneau J, Landier J, et al. Mycobacterium ulcerans ecological dynamics and its association with freshwater ecosystems and aquatic communities: results from a 12-month environmental survey in Cameroon. PLoS neglected tropical diseases. 2014;8(5):e2879.
18. Marion E, Eyangoh S, Yeramian E, Doannio J, Landier J, Aubry J, et al. Seasonal and regional dynamics of M. ulcerans transmission in environmental context: deciphering the role of water bugs as hosts and vectors. PLoS neglected tropical diseases. 2010;4(7):e731.
19. Konan KL, Doannio JM, Coulibaly NG, Ekaza E, Marion E, Asse H, et al. [Detection of the IS2404 insertion sequence and ketoreductase produced by Mycobacterium ulcerans in the aquatic Heteroptera in the health districts of Dabou and Tiassale in Cote d'Ivoire]. Medecine et sante tropicales. 2015;25(1):44-51.
20. Tano MB, Dassi C, Mosi L, Koussemon M, Bonfoh B. Molecular Characterization of Mycolactone Producing Mycobacteria from Aquatic Environments in Buruli Ulcer Non-Endemic Areas in Cote d'Ivoire. Int J Environ Res Public Health. 2017;14(2).
21. Morris A, Gozlan R, Marion E, Marsollier L, Andreou D, Sanhueza D, et al. First Detection of Mycobacterium ulcerans DNA in Environmental Samples from South America. PLoS neglected tropical diseases. 2014;8(1).

22. Aboagye SY, Ampah KA, Ross A, Asare P, Otchere ID, Fyfe J, et al. Seasonal Pattern of Mycobacterium ulcerans, the Causative Agent of Buruli Ulcer, in the Environment in Ghana. Microb Ecol. 2017;74(2):350-61.
23. Amissah NA, Gryseels S, Tobias NJ, Ravadgar B, Suzuki M, Vandelannoote K, et al. Investigating the role of free-living amoebae as a reservoir for Mycobacterium ulcerans. PLoS neglected tropical diseases. 2014;8(9):e3148.
24. Benbow ME, Kimbirauskas R, McIntosh MD, Williamson H, Quaye C, Boakye D, et al. Aquatic macroinvertebrate assemblages of Ghana, West Africa: understanding the ecology of a neglected tropical disease. EcoHealth. 2014;11(2):168-83.
25. Narh CA, Mosi L, Quaye C, Dassi C, Konan DO, Tay SC, et al. Source tracking Mycobacterium ulcerans infections in the Ashanti region, Ghana. PLoS neglected tropical diseases. 2015;9(1):e0003437.
26. Tobias NJ, Ammisah NA, Ahortor EK, Wallace JR, Ablordey A, Stinear TP. Snapshot fecal survey of domestic animals in rural Ghana for Mycobacterium ulcerans. PeerJ. 2016;4:e2065.
27. Vandelannoote K, Durnez L, Amissah D, Gryseels S, Dodoo A, Yeboah S, et al. Application of real-time PCR in Ghana, a Buruli ulcer-endemic country, confirms the presence of Mycobacterium ulcerans in the environment. FEMS Microbiol Lett. 2010;304(2):191-4.
28. Benbow ME, Williamson H, Kimbirauskas R, McIntosh MD, Kolar R, Quaye C, et al. Aquatic invertebrates as unlikely vectors of Buruli ulcer disease. Emerging infectious diseases. 2008;14(8):1247-54.
29. Willson SJ, Kaufman MG, Merritt RW, Williamson HR, Malakauskas DM, Benbow ME. Fish and amphibians as potential reservoirs of Mycobacterium ulcerans, the causative agent of Buruli ulcer disease. Infection ecology & epidemiology. 2013;3.
30. Ohtsuka M, Kikuchi N, Yamamoto T, Suzutani T, Nakanaga K, Suzuki K, et al. Buruli ulcer caused by Mycobacterium ulcerans subsp shinshuense: a rare case of familial concurrent occurrence and detection of insertion sequence 2404 in Japan. JAMA dermatology. 2014;150(1):64-7.
31. Maman I, Tchacondo T, Kere AB, Beissner M, Badziklou K, Tedihou E, et al. Molecular detection of Mycobacterium ulcerans in the environment and its relationship with Buruli ulcer occurrence in Zio and Yoto districts of maritime region in Togo. PLoS neglected tropical diseases. 2018;12(5):e0006455.
32. Hennigan CE, Myers L, Ferris MJ. Environmental Distribution and Seasonal Prevalence of Mycobacterium ulcerans in Southern Louisiana. Applied and environmental microbiology. 2013;79(8):2648-56.

Table S.5: results of evidence consensus framework showing strength of evidence for BU presence or absence worldwide.

Literature review scores D, C, and DQ show the scores assigned to the highest-scoring reference, based on diagnostic specificity (D) and contemporariness (C), combined to give a data quality (DQ) score. The total cases (TC) is the sum of the total number of cases reported in all references included in the review, each weighted according to the data quality score for the reference. Health expenditure level (HE L) was used to assign health system (HS) scores 1 and 2. Misdiagnosis scores were calculated for a range of conditions that present on the skin, based on the proportion of symptoms shared with BU. The composite misdiagnosis likelihood score (CMP) is the sum of the misdiagnosis scores for all endemic diseases in each country, weighted by HS1 which represents the quality of diagnosis. The evidence consensus score (ECS) is the sum of columns in bold. Positive values of ECS indicate evidence for presence, negative values indicate evidence for absence.

	Health reporting organisations				Literature review						Health expenditure			Misdiagnosis likelihood scores							ECS
NAME	WHO rep	WHO score	GIDEON score	**HRO score**	Highest scoring reference	D	C	**DQ**	TC	**CS**	HE L	HS 1	**HS 2**	YW	CL	ONC	LPR	LF	TU	**CMP**	
Australia	886	1.5	0.5	**2**	Tai, A. et al. (2018) (1)	1.0	1	**2.0**	1138	**1**	H	0.25	**0**				0.002			**0.00**	100
Benin	5534	1.5	0.5	**2**	Sopoh, G. et al. (2010) (2)	1.0	1	**2.0**	8629	**1**	L	1	**1**				0.002	0.005	0.71	**0.56**	100
Cameroon	2045	1.5	0.5	**2**	Landier, J. et al. (2011) (3)	1.0	1	**2.0**	5273	**1**	L	1	**1**	0.16	0.35	0.06	0.002	0.005	0.71	**1.00**	100
Côte d'Ivoire	15481	1.5	0.5	**2**	N'Krumah, R. et al. (2017) (4)	1.0	1	**2.0**	3664	**1**	L	1	**1**	0.16	0.35		0.002			**0.40**	100
Democratic Republic of Congo	2216	1.5	0.5	**2**	Mavinga Phanzu, D. et al. (2011) (5)	1.0	1	**2.0**	813	**1**	L	1	**1**	0.16		0.06		0.005		**0.17**	100
Gabon	483	1.5	0.5	**2**	Bayonne Manou, L. S. et al. (2013) (6)	1.0	1	**2.0**	295	**1**	M	0.5	**0.5**			0.06	0.002	0.005	0.71	**0.30**	100
Ghana	6797	1.5	0.5	**2**	Yeboah-Manu, D. et al. (2018) (7)	1.0	1	**2.0**	4932	**1**	L	1	**1**	0.16	0.35		0.002	0.005	0.71	**0.96**	100
Japan	56	1.5	0.5	**2**	Nakanaga, K. et al. (2013) (8)	1.0	1	**2.0**	38	**1**	H	0.25	**0**							**0.00**	100
Liberia	134	1.5	0.5	**2**	Kollie, K. et al. (2014) (9)	1.0	1	**2.0**	25	**1**	L	1	**1**			0.06		0.005	0.71	**0.60**	100
Nigeria	512	1.5	0.5	**2**	Ayelo, G. A. et al. (2018) (10)	1.0	1	**2.0**	600	**1**	L	1	**1**	0.16	0.35	0.06	0.002	0.005	0.71	**1.00**	100
Togo	726	1.5	0.5	**2**	Beissner, M. et al. (2015) (11)	1.0	1	**2.0**	373	**1**	L	1	**1**				0.002	0.005	0.71	**0.56**	100
South Sudan	20	1.5	0.5	**2**	South Sudan MoH (2015) (12)	1.0	1	**2.0**	626	**1**	L	1	**1**		0.35	0.06		0.005		**0.32**	100
Republic of Congo	579	1.5	0.5	**2**	Marion, E., et al. (2014 b) (13)	1.0	1	**2.0**	12	**0.75**	M	0.5	**0.5**	0.16		0.06	0.002	0.005	0.71	**0.36**	95
Sierra Leone	29	1.5	0.5	**2**	Murphy, H. E. (2013) (14)	1.0	1	**2.0**	17	**0.75**	L	1	**1**					0.005	0.71	**0.56**	95
CAR	3	1.5	0.5	**2**	Minime-Lingoupou, F. et al. (2010) (15)	1.0	1	**2.0**	2	**0.25**	L	1	**1**	0.16		0.06		0.005	0.71	**0.73**	85
PNG	85	1.5	0.5	**2**	Igo, et al. (1988) (16)	0.6	0.5	**1.1**	239	**1**	L	1	**1**	0.16				0.005	0.71	**0.68**	83
Guinea	520	1.5	0.5	**2**	WHO (2001) (Sagno) (17)	0.5	0.5	**1.0**	111	**1**	L	1	**1**				0.002	0.005	0.71	**0.56**	80
Uganda	58	1.5	0.5	**2**	Bradley, D. et al (1970) (18)	0.6	0.25	**0.9**	123	**1**	L	1	**1**			0.06	0.002	0.005	0.71	**0.60**	78

NAME	Health reporting organisations				Literature review						Health expenditure			Misdiagnosis likelihood scores							ECS
	WHO rep	WHO score	GIDEON score	**HRO score**	Highest scoring reference		D	C	**DQ**	TC	**CS**	HE L	HS 1	**HS 2**	YW	CL	ONC	LPR	LF	TU	**CMP**	
French Guiana	H	-1	0.5	**-0.5**	Reynaud, Y. et al. (2015)	(19)	1.0	1	**2.0**	35	**1**	H	0.25	**0**		0.35				0.71	**0.21**	50
Kenya	H	-1	0.5	**-0.5**	Walsh, D. S. et al. (2009)	(20)	1.0	1	**2.0**	1	**0.25**	L	1	**1**		0.35	0.06		0.005	0.71	**0.87**	35
Brazil	H	-1	0.5	**-0.5**	dos Santos, J. (2007)	(21)	1.0	1	**2.0**	1	**0.25**	H	0.25	**0**		0.35	0.06	0.002	0.005	0.71	**0.22**	35
Peru	0	-1.5	0.5	**-1**	Guerra, H. et al. (2008)	(22)	1.0	1	**2.0**	9	**0.5**	M	0.5	**0.5**		0.35		0.002		0.71	**0.41**	30
Angola	0	-1.5	0.5	**-1**	Kibadi, K. et al. (2008)	(23)	1.0	1	**2.0**	3	**0.25**	M	0.5	**0.5**			0.06		0.005	0.71	**0.30**	25
Jordan	0	-1.5	0.5	**-1**	Al Ramahi et al. (2017)	(24)	1.0	1	**2.0**	1	**0.25**	M	0.5	**0.5**		0.35					**0.14**	25
Mali	0	-1.5	0.5	**-1**	Bessis, D. et al (2015)	(25)	1.0	1	**2.0**	3	**0.25**	L	1	**1**		0.35		0.002	0.005	0.71	**0.83**	25
Mexico	H	-1	0.5	**-0.5**	Coloma, J. N. et al. (2005)	(26)	0.8	0.5	**1.3**	2	**0.25**	H	0.25	**0**		0.35		0.002			**0.07**	20
China	H	-1	0.5	**-0.5**	Faber, W. R. et al. (2000)	(27)	0.8	0.5	**1.3**	1	**0.25**	M	0.5	**0.5**		0.35		0.002			**0.14**	20
Malawi	H	-1	0.5	**-0.5**	Komolafe, O. O. (2001)	(28)	0.7	0.5	**1.2**	2	**0.25**	L	1	**1**		0.35	0.06		0.005	0.71	**0.87**	20
Suriname	H	-1	0.5	**-0.5**	Faber, W. R. et al. (2015)	(29)	0.6	0.25	**0.9**	1	**0.25**	H	0.25	**0**		0.35		0.002	0.005	0.71	**0.21**	13
Burkina Faso	0	-1.5	0.5	**-1**	Ouoba, K., et al. (1998)	(30)	0.8	0.5	**1.3**	2	**0.25**	L	1	**1**		0.35		0.002	0.005	0.71	**0.83**	10
Honduras	0	-1.5	-0.5	**-2**	Southern, Paul M. (2016)	(31)	1.0	1	**2.0**	1	**0.25**	M	0.5	**0.5**		0.35				0.71	**0.41**	5
Ethiopia	0	-1.5	-0.5	**-2**	Gordon, D. et al. (2014)	(32)	1.0	1	**2.0**	2	**0.25**	L	1	**1**		0.35	0.06	0.002	0.005	0.71	**0.87**	5
Malaysia	H	-1	0.5	**-0.5**	Stanford et al. (1973)	(33)	0.0	0	**0.5***|		M	0.5	**0.5**				0.002	0.005	0.71	**0.28**	0
Niger	0	-1.5	-0.5	**-2**								L	1	**1**		0.35		0.002	0.005	0.71	**0.83**	-9
Eritrea	0	-1.5	-0.5	**-2**								L	1	**1**		0.35			0.005	0.71	**0.83**	-9
Gambia	0	-1.5	-0.5	**-2**								L	1	**1**		0.35			0.005	0.71	**0.83**	-9
Mauritania	0	-1.5	-0.5	**-2**								L	1	**1**		0.35				0.71	**0.82**	-9
Indonesia	H	-1	0.5	**-0.5**								M	0.5	**0.5**	0.16			0.002	0.005	0.71	**0.34**	-10
Equatorial Guinea	H	-1	0.5	**-0.5**								M	0.5	**0.5**			0.06	0.002	0.005	0.71	**0.30**	-10
Kiribati	H	-1	0.5	**-0.5**								M	0.5	**0.5**				0.002	0.005		**0.00**	-10
Sri Lanka	H	-1	0.5	**-0.5**								L	1	**1**		0.35		0.002	0.005		**0.28**	-10
Mozambique	0	-1.5	-0.5	**-2**								L	1	**1**			0.06	0.002	0.005	0.71	**0.60**	-20
Rwanda	0	-1.5	-0.5	**-2**								L	1	**1**			0.06	0.002	0.005	0.71	**0.60**	-20
Senegal	0	-1.5	0.5	**-1**								L	1	**1**		0.35		0.002	0.005	0.71	**0.83**	-20

NAME	Health reporting organisations				Literature review						Health expenditure			Misdiagnosis likelihood scores							ECS
	WHO rep	WHO score	GIDEON score	**HRO score**	Highest scoring reference	D	C	**DQ**	TC	**CS**	HE L	HS 1	**HS 2**	YW	CL	ONC	LPR	LF	TU	**CMP**	
Burundi	0	-1.5	-0.5	**-2**							L	1	**1**			0.06		0.005	0.71	**0.60**	-20
Chad	0	-1.5	-0.5	**-2**							L	1	**1**			0.06		0.005	0.71	**0.60**	-20
Madagascar	0	-1.5	-0.5	**-2**							L	1	**1**				0.002	0.005	0.71	**0.56**	-22
Guinea-Bissau	0	-1.5	-0.5	**-2**							L	1	**1**					0.005	0.71	**0.56**	-22
Sao Tome and Principe	0	-1.5	-0.5	**-2**							L	1	**1**					0.005	0.71	**0.56**	-22
Zambia	0	-1.5	-0.5	**-2**							L	1	**1**					0.005	0.71	**0.56**	-22
Zimbabwe	0	-1.5	-0.5	**-2**							L	1	**1**					0.005	0.71	**0.56**	-22
Somalia	0	-1.5	-0.5	**-2**							L	1	**1**				0.002		0.71	**0.55**	-22
Iran, Islamic Republic of	0	-1.5	-0.5	-2	Behrouznasab et al. (2012) (34)			**0.5***			H	0.25	**0**		0.35					**0.07**	-29
Timor-Leste	0	-1.5	-0.5	**-2**							L	1	**1**	0.16	0.35		0.002	0.005		**0.40**	-30
India	0	-1.5	-0.5	**-2**							L	1	**1**		0.35		0.002	0.005		**0.28**	-36
Nepal	0	-1.5	-0.5	**-2**							L	1	**1**		0.35		0.002	0.005		**0.28**	-36
Yemen	0	-1.5	-0.5	**-2**							L	1	**1**		0.35		0.002	0.005		**0.28**	-36
Afghanistan	0	-1.5	-0.5	**-2**							L	1	**1**		0.35		0.002			**0.27**	-36
Pakistan	0	-1.5	-0.5	**-2**							L	1	**1**		0.35		0.002			**0.27**	-36
Syrian Arab Republic	0	-1.5	-0.5	**-2**							L	1	**1**		0.35		0.002			**0.27**	-36
Uzbekistan	0	-1.5	-0.5	**-2**							L	1	**1**		0.35					**0.27**	-36
West Bank	0	-1.5	-0.5	**-2**							L	1	**1**		0.35					**0.27**	-36
United Republic of Tanzania	0	-1.5	-0.5	**-2**							L	1	**1**			0.06		0.005		**0.05**	-48
Bangladesh	0	-1.5	-0.5	**-2**							L	1	**1**				0.002	0.005		**0.01**	-50
Comoros	0	-1.5	-0.5	**-2**							L	1	**1**				0.002	0.005		**0.01**	-50
Haiti	0	-1.5	-0.5	**-2**							L	1	**1**				0.002	0.005		**0.01**	-50
Myanmar	0	-1.5	-0.5	**-2**							L	1	**1**				0.002	0.005		**0.01**	-50
Viet Nam	0	-1.5	-0.5	**-2**							L	1	**1**				0.002	0.005		**0.01**	-50
Cambodia	0	-1.5	-0.5	**-2**							L	1	**1**					0.005		**0.00**	-50

NAME	Health reporting organisations				Literature review						Health expenditure			Misdiagnosis likelihood scores							ECS
	WHO rep	WHO score	GIDEON score	**HRO score**	Highest scoring reference	D	C	**DQ**	TC	**CS**	HE L	HS 1	**HS 2**	YW	CL	ONC	LPR	LF	TU	**CMP**	
Lao People's Democratic Republic	0	-1.5	-0.5	**-2**							L	1	**1**					0.005		**0.00**	-50
Bhutan	0	-1.5	-0.5	**-2**							L	1	**1**							**0.00**	-50
Dem People's Rep of Korea	0	-1.5	-0.5	**-2**							L	1	**1**							**0.00**	-50
Djibouti	0	-1.5	-0.5	**-2**							L	1	**1**							**0.00**	-50
Kyrgyzstan	0	-1.5	-0.5	**-2**							L	1	**1**							**0.00**	-50
Tajikistan	0	-1.5	-0.5	**-2**							L	1	**1**							**0.00**	-50
Guyana	0	-1.5	-0.5	**-2**							M	0.5	**0.5**	0.16	0.35		0.002	0.005	0.71	**0.48**	-51
Ecuador	0	-1.5	-0.5	**-2**							M	0.5	**0.5**	0.16	0.35				0.71	**0.48**	-51
Sudan	0	-1.5	-0.5	**-2**							M	0.5	**0.5**		0.35	0.06	0.002	0.005	0.71	**0.44**	-53
Thailand	0	-1.5	-0.5	**-2**							M	0.5	**0.5**		0.35		0.002	0.005	0.71	**0.41**	-54
Colombia	0	-1.5	-0.5	**-2**							M	0.5	**0.5**		0.35		0.002		0.71	**0.41**	-54
Algeria	0	-1.5	-0.5	**-2**							M	0.5	**0.5**		0.35				0.71	**0.41**	-54
Belize	0	-1.5	-0.5	**-2**							M	0.5	**0.5**		0.35				0.71	**0.41**	-54
Bolivia	0	-1.5	-0.5	**-2**							M	0.5	**0.5**		0.35				0.71	**0.41**	-54
El Salvador	0	-1.5	-0.5	**-2**							M	0.5	**0.5**		0.35				0.71	**0.41**	-54
Guatemala	0	-1.5	-0.5	**-2**							M	0.5	**0.5**		0.35				0.71	**0.41**	-54
Nicaragua	0	-1.5	-0.5	**-2**							M	0.5	**0.5**		0.35				0.71	**0.41**	-54
Philippines	0	-1.5	-0.5	**-2**							M	0.5	**0.5**				0.002	0.005	0.71	**0.28**	-61
Botswana	0	-1.5	-0.5	**-2**							M	0.5	**0.5**						0.71	**0.28**	-61
Swaziland	0	-1.5	-0.5	**-2**							M	0.5	**0.5**						0.71	**0.28**	-61
Egypt	0	-1.5	-0.5	**-2**							M	0.5	**0.5**		0.35		0.002	0.005		**0.14**	-68
Dominican Republic	0	-1.5	-0.5	**-2**							M	0.5	**0.5**		0.35			0.005		**0.14**	-68
Morocco	0	-1.5	-0.5	**-2**							M	0.5	**0.5**		0.35		0.002			**0.14**	-68
Paraguay	0	-1.5	-0.5	**-2**							M	0.5	**0.5**		0.35		0.002			**0.14**	-68
Azerbaijan	0	-1.5	-0.5	**-2**							M	0.5	**0.5**		0.35					**0.14**	-68
Georgia	0	-1.5	-0.5	**-2**							M	0.5	**0.5**		0.35					**0.14**	-68
Iraq	0	-1.5	-0.5	**-2**							M	0.5	**0.5**		0.35					**0.14**	-68

NAME	Health reporting organisations				Literature review						Health expenditure			Misdiagnosis likelihood scores							ECS
	WHO rep	WHO score	GIDEON score	**HRO score**	Highest scoring reference	D	C	**DQ**	TC	**CS**	HE L	HS 1	**HS 2**	YW	CL	ONC	LPR	LF	TU	**CMP**	
Tunisia	0	-1.5	-0.5	**-2**							M	0.5	**0.5**		0.35					**0.14**	-68
Turkmenistan	0	-1.5	-0.5	**-2**							M	0.5	**0.5**		0.35					**0.14**	-68
Vanuatu	0	-1.5	-0.5	**-2**							M	0.5	**0.5**	0.16				0.005		**0.07**	-72
Seychelles	0	-1.5	-0.5	**-2**							M	0.5	**0.5**				0.002	0.005		**0.00**	-75
Solomon Islands	0	-1.5	-0.5	**-2**							M	0.5	**0.5**				0.002	0.005		**0.00**	-75
Cape Verde	0	-1.5	-0.5	**-2**							M	0.5	**0.5**					0.005		**0.00**	-75
Cook Islands	0	-1.5	-0.5	**-2**							M	0.5	**0.5**					0.005		**0.00**	-75
Fiji	0	-1.5	-0.5	**-2**							M	0.5	**0.5**					0.005		**0.00**	-75
Mauritius	0	-1.5	-0.5	**-2**							M	0.5	**0.5**					0.005		**0.00**	-75
Samoa	0	-1.5	-0.5	**-2**							M	0.5	**0.5**					0.005		**0.00**	-75
Tonga	0	-1.5	-0.5	**-2**							M	0.5	**0.5**					0.005		**0.00**	-75
Tuvalu	0	-1.5	-0.5	**-2**							M	0.5	**0.5**					0.005		**0.00**	-75
Albania	0	-1.5	-0.5	**-2**							M	0.5	**0.5**							**0.00**	-75
Armenia	0	-1.5	-0.5	**-2**							M	0.5	**0.5**							**0.00**	-75
Belarus	0	-1.5	-0.5	**-2**							M	0.5	**0.5**							**0.00**	-75
Bosnia and Herzegovina	0	-1.5	-0.5	**-2**							M	0.5	**0.5**							**0.00**	-75
Dominica	0	-1.5	-0.5	**-2**							M	0.5	**0.5**							**0.00**	-75
Grenada	0	-1.5	-0.5	**-2**							M	0.5	**0.5**							**0.00**	-75
Jamaica	0	-1.5	-0.5	**-2**							M	0.5	**0.5**							**0.00**	-75
Kazakhstan	0	-1.5	-0.5	**-2**							M	0.5	**0.5**							**0.00**	-75
Kingman Reef	0	-1.5	-0.5	**-2**							M	0.5	**0.5**							**0.00**	-75
Lesotho	0	-1.5	-0.5	**-2**							M	0.5	**0.5**							**0.00**	-75
Mongolia	0	-1.5	-0.5	**-2**							M	0.5	**0.5**							**0.00**	-75
Montenegro	0	-1.5	-0.5	**-2**							M	0.5	**0.5**							**0.00**	-75
Paracel Islands	0	-1.5	-0.5	**-2**							M	0.5	**0.5**							**0.00**	-75
Republic of Moldova	0	-1.5	-0.5	**-2**							M	0.5	**0.5**							**0.00**	-75
Romania	0	-1.5	-0.5	**-2**							M	0.5	**0.5**							**0.00**	-75
Saint Lucia	0	-1.5	-0.5	**-2**							M	0.5	**0.5**							**0.00**	-75

NAME	Health reporting organisations				Literature review						Health expenditure			Misdiagnosis likelihood scores							ECS
	WHO rep	WHO score	GIDEON score	**HRO score**	Highest scoring reference	D	C	**DQ**	TC	**CS**	HE L	HS 1	**HS 2**	YW	CL	ONC	LPR	LF	TU	**CMP**	
Saint Vincent and the Grenadines	0	-1.5	-0.5	**-2**							M	0.5	**0.5**							**0.00**	-75
Spratly Islands	0	-1.5	-0.5	**-2**							M	0.5	**0.5**							**0.00**	-75
Ukraine	0	-1.5	-0.5	**-2**							M	0.5	**0.5**							**0.00**	-75
Western Sahara	0	-1.5	-0.5	**-2**							M	0.5	**0.5**							**0.00**	-75
Venezuela	0	-1.5	-0.5	**-2**							H	0.25	**0**		0.35	0.06		0.005	0.71	**0.22**	-89
Costa Rica	0	-1.5	-0.5	**-2**							H	0.25	**0**		0.35		0.002	0.005	0.71	**0.21**	-90
Argentina	0	-1.5	-0.5	**-2**							H	0.25	**0**		0.35		0.002		0.71	**0.21**	-90
Namibia	0	-1.5	-0.5	**-2**							H	0.25	**0**		0.35				0.71	**0.21**	-90
Panama	0	-1.5	-0.5	**-2**							H	0.25	**0**		0.35				0.71	**0.21**	-90
South Africa	0	-1.5	-0.5	**-2**							H	0.25	**0**				0.002		0.71	**0.14**	-93
Chile	0	-1.5	-0.5	**-2**							H	0.25	**0**						0.71	**0.14**	-93
Saint Helena	0	-1.5	-0.5	**-2**							H	0.25	**0**						0.71	**0.14**	-93
Croatia	0	-1.5	-0.5	**-2**							H	0.25	**0**		0.35					**0.07**	-97
Cyprus	0	-1.5	-0.5	**-2**							H	0.25	**0**		0.35					**0.07**	-97
France	0	-1.5	-0.5	**-2**							H	0.25	**0**		0.35					**0.07**	-97
Greece	0	-1.5	-0.5	**-2**							H	0.25	**0**		0.35					**0.07**	-97
Israel	0	-1.5	-0.5	**-2**							H	0.25	**0**		0.35					**0.07**	-97
Italy	0	-1.5	-0.5	**-2**							H	0.25	**0**		0.35					**0.07**	-97
Kuwait	0	-1.5	-0.5	**-2**							H	0.25	**0**		0.35					**0.07**	-97
Lebanon	0	-1.5	-0.5	**-2**							H	0.25	**0**		0.35					**0.07**	-97
Libyan Arab Jamahiriya	0	-1.5	-0.5	**-2**							H	0.25	**0**		0.35					**0.07**	-97
Malta	0	-1.5	-0.5	**-2**							H	0.25	**0**		0.35					**0.07**	-97
Martinique	0	-1.5	-0.5	**-2**							H	0.25	**0**		0.35					**0.07**	-97
Portugal	0	-1.5	-0.5	**-2**							H	0.25	**0**		0.35					**0.07**	-97
Saudi Arabia	0	-1.5	-0.5	**-2**							H	0.25	**0**		0.35					**0.07**	-97
Spain	0	-1.5	-0.5	**-2**							H	0.25	**0**		0.35					**0.07**	-97
Turkey	0	-1.5	-0.5	**-2**							H	0.25	**0**		0.35					**0.07**	-97

NAME	Health reporting organisations				Literature review						Health expenditure			Misdiagnosis likelihood scores							ECS
	WHO rep	WHO score	GIDEON score	**HRO score**	Highest scoring reference	D	C	**DQ**	TC	**CS**	HE L	HS 1	**HS 2**	YW	CL	ONC	LPR	LF	TU	**CMP**	
USA	0	-1.5	-0.5	**-2**							H	0.25	**0**		0.35					**0.07**	-97
Marshall Islands	0	-1.5	-0.5	**-2**							H	0.25	**0**				0.002	0.005		**0.00**	-100
New Caledonia	0	-1.5	-0.5	**-2**							H	0.25	**0**				0.002	0.005		**0.00**	-100
Trinidad and Tobago	0	-1.5	-0.5	**-2**							H	0.25	**0**				0.002	0.005		**0.00**	-100
American Samoa	0	-1.5	-0.5	**-2**							H	0.25	**0**					0.005		**0.00**	-100
French Polynesia	0	-1.5	-0.5	**-2**							H	0.25	**0**					0.005		**0.00**	-100
Maldives	0	-1.5	-0.5	**-2**							H	0.25	**0**					0.005		**0.00**	-100
Niue	0	-1.5	-0.5	**-2**							H	0.25	**0**					0.005		**0.00**	-100
Palau	0	-1.5	-0.5	**-2**							H	0.25	**0**					0.005		**0.00**	-100
Wallis and Futuna	0	-1.5	-0.5	**-2**							H	0.25	**0**					0.005		**0.00**	-100
Bahamas	0	-1.5	-0.5	**-2**							H	0.25	**0**				0.002			**0.00**	-100
Cuba	0	-1.5	-0.5	**-2**							H	0.25	**0**				0.002			**0.00**	-100
Qatar	0	-1.5	-0.5	**-2**							H	0.25	**0**				0.002			**0.00**	-100
Andorra	0	-1.5	-0.5	**-2**							H	0.25	**0**							**0.00**	-100
Anguilla	0	-1.5	-0.5	**-2**							H	0.25	**0**							**0.00**	-100
Antarctica	0	-1.5	-0.5	**-2**							H	0.25	**0**							**0.00**	-100
Antigua and Barbuda	0	-1.5	-0.5	**-2**							H	0.25	**0**							**0.00**	-100
Aruba	0	-1.5	-0.5	**-2**							H	0.25	**0**							**0.00**	-100
Austria	0	-1.5	-0.5	**-2**							H	0.25	**0**							**0.00**	-100
Bahrain	0	-1.5	-0.5	**-2**							H	0.25	**0**							**0.00**	-100
Baker Island	0	-1.5	-0.5	**-2**							H	0.25	**0**							**0.00**	-100
Barbados	0	-1.5	-0.5	**-2**							H	0.25	**0**							**0.00**	-100
Belgium	0	-1.5	-0.5	**-2**							H	0.25	**0**							**0.00**	-100
Bermuda	0	-1.5	-0.5	**-2**							H	0.25	**0**							**0.00**	-100
Bouvet Island	0	-1.5	-0.5	**-2**							H	0.25	**0**							**0.00**	-100
British Indian Ocean Territory	0	-1.5	-0.5	**-2**							H	0.25	**0**							**0.00**	-100

NAME	Health reporting organisations				Literature review						Health expenditure			Misdiagnosis likelihood scores							ECS
	WHO rep	WHO score	GIDEON score	**HRO score**	Highest scoring reference	D	C	**DQ**	TC	**CS**	HE L	HS 1	**HS 2**	YW	CL	ONC	LPR	LF	TU	**CMP**	
British Virgin Islands	0	-1.5	-0.5	**-2**							H	0.25	**0**							**0.00**	-100
Bulgaria	0	-1.5	-0.5	**-2**							H	0.25	**0**							**0.00**	-100
Canada	0	-1.5	-0.5	**-2**							H	0.25	**0**							**0.00**	-100
Cayman Islands	0	-1.5	-0.5	**-2**							H	0.25	**0**							**0.00**	-100
Christmas Island	0	-1.5	-0.5	**-2**							H	0.25	**0**							**0.00**	-100
Clipperton Island	0	-1.5	-0.5	**-2**							H	0.25	**0**							**0.00**	-100
Cocos (Keeling) Islands	0	-1.5	-0.5	**-2**							H	0.25	**0**							**0.00**	-100
Czech Republic	0	-1.5	-0.5	**-2**							H	0.25	**0**							**0.00**	-100
Denmark	0	-1.5	-0.5	**-2**							H	0.25	**0**							**0.00**	-100
Estonia	0	-1.5	-0.5	**-2**							H	0.25	**0**							**0.00**	-100
Europa Island	0	-1.5	-0.5	**-2**							H	0.25	**0**							**0.00**	-100
Falkland Islands (Malvinas)	0	-1.5	-0.5	**-2**							H	0.25	**0**							**0.00**	-100
Faroe Islands	0	-1.5	-0.5	**-2**							H	0.25	**0**							**0.00**	-100
Finland	0	-1.5	-0.5	**-2**							H	0.25	**0**							**0.00**	-100
French Southern and Antarctic Territories	0	-1.5	-0.5	**-2**							H	0.25	**0**							**0.00**	-100
Germany	0	-1.5	-0.5	**-2**							H	0.25	**0**							**0.00**	-100
Gibraltar	0	-1.5	-0.5	**-2**							H	0.25	**0**							**0.00**	-100
Greenland	0	-1.5	-0.5	**-2**							H	0.25	**0**							**0.00**	-100
Guadeloupe	0	-1.5	-0.5	**-2**							H	0.25	**0**							**0.00**	-100
Guam	0	-1.5	-0.5	**-2**							H	0.25	**0**							**0.00**	-100
Guernsey	0	-1.5	-0.5	**-2**							H	0.25	**0**							**0.00**	-100
Heard Island and McDonald Islands	0	-1.5	-0.5	**-2**							H	0.25	**0**							**0.00**	-100

	Health reporting organisations				Literature review						Health expenditure			Misdiagnosis likelihood scores							ECS
NAME	WHO rep	WHO score	GIDEON score	**HRO score**	Highest scoring reference	D	C	**DQ**	TC	**CS**	HE L	HS 1	**HS 2**	YW	CL	ONC	LPR	LF	TU	**CMP**	
Holy See	0	-1.5	-0.5	**-2**							H	0.25	**0**							**0.00**	-100
Hong Kong	0	-1.5	-0.5	**-2**							H	0.25	**0**							**0.00**	-100
Hungary	0	-1.5	-0.5	**-2**							H	0.25	**0**							**0.00**	-100
Iceland	0	-1.5	-0.5	**-2**							H	0.25	**0**							**0.00**	-100
Ireland	0	-1.5	-0.5	**-2**							H	0.25	**0**							**0.00**	-100
Isle of Man	0	-1.5	-0.5	**-2**							H	0.25	**0**							**0.00**	-100
Jersey	0	-1.5	-0.5	**-2**							H	0.25	**0**							**0.00**	-100
Korea, Republic of	0	-1.5	-0.5	**-2**							H	0.25	**0**							**0.00**	-100
Latvia	0	-1.5	-0.5	**-2**							H	0.25	**0**							**0.00**	-100
Liechtenstein	0	-1.5	-0.5	**-2**							H	0.25	**0**							**0.00**	-100
Lithuania	0	-1.5	-0.5	**-2**							H	0.25	**0**							**0.00**	-100
Luxembourg	0	-1.5	-0.5	**-2**							H	0.25	**0**							**0.00**	-100
Macau	0	-1.5	-0.5	**-2**							H	0.25	**0**							**0.00**	-100
Mayotte	0	-1.5	-0.5	**-2**							H	0.25	**0**							**0.00**	-100
Monaco	0	-1.5	-0.5	**-2**							H	0.25	**0**							**0.00**	-100
Montserrat	0	-1.5	-0.5	**-2**							H	0.25	**0**							**0.00**	-100
Nauru	0	-1.5	-0.5	**-2**							H	0.25	**0**							**0.00**	-100
Netherlands	0	-1.5	-0.5	**-2**							H	0.25	**0**							**0.00**	-100
Netherlands Antilles	0	-1.5	-0.5	**-2**							H	0.25	**0**							**0.00**	-100
New Zealand	0	-1.5	-0.5	**-2**							H	0.25	**0**							**0.00**	-100
Norfolk Island	0	-1.5	-0.5	**-2**							H	0.25	**0**							**0.00**	-100
Northern Mariana Islands	0	-1.5	-0.5	**-2**							H	0.25	**0**							**0.00**	-100
Norway	0	-1.5	-0.5	**-2**							H	0.25	**0**							**0.00**	-100
Oman	0	-1.5	-0.5	**-2**							H	0.25	**0**							**0.00**	-100
Pitcairn	0	-1.5	-0.5	**-2**							H	0.25	**0**							**0.00**	-100
Poland	0	-1.5	-0.5	**-2**							H	0.25	**0**							**0.00**	-100
Puerto Rico	0	-1.5	-0.5	**-2**							H	0.25	**0**							**0.00**	-100
Reunion	0	-1.5	-0.5	**-2**							H	0.25	**0**							**0.00**	-100

NAME	Health reporting organisations				Literature review						Health expenditure			Misdiagnosis likelihood scores							ECS
	WHO rep	WHO score	GIDEON score	**HRO score**	Highest scoring reference	D	C	**DQ**	TC	**CS**	HE L	HS 1	**HS 2**	YW	CL	ONC	LPR	LF	TU	**CMP**	
Russian Federation	0	-1.5	-0.5	**-2**							H	0.25	**0**							**0.00**	-100
Saint Kitts and Nevis	0	-1.5	-0.5	**-2**							H	0.25	**0**							**0.00**	-100
Saint Pierre et Miquelon	0	-1.5	-0.5	**-2**							H	0.25	**0**							**0.00**	-100
San Marino	0	-1.5	-0.5	**-2**							H	0.25	**0**							**0.00**	-100
Serbia	0	-1.5	-0.5	**-2**							H	0.25	**0**							**0.00**	-100
Singapore	0	-1.5	-0.5	**-2**							H	0.25	**0**							**0.00**	-100
Slovakia	0	-1.5	-0.5	**-2**							H	0.25	**0**							**0.00**	-100
Slovenia	0	-1.5	-0.5	**-2**							H	0.25	**0**							**0.00**	-100
South Georgia and the South Sandwich Islands	0	-1.5	-0.5	**-2**							H	0.25	**0**							**0.00**	-100
Svalbard and Jan Mayen Islands	0	-1.5	-0.5	**-2**							H	0.25	**0**							**0.00**	-100
Sweden	0	-1.5	-0.5	**-2**							H	0.25	**0**							**0.00**	-100
Switzerland	0	-1.5	-0.5	**-2**							H	0.25	**0**							**0.00**	-100
Tokelau	0	-1.5	-0.5	**-2**							H	0.25	**0**							**0.00**	-100
Turks and Caicos islands	0	-1.5	-0.5	**-2**							H	0.25	**0**							**0.00**	-100
U.K.	0	-1.5	-0.5	**-2**							H	0.25	**0**							**0.00**	-100
UAE	0	-1.5	-0.5	**-2**							H	0.25	**0**							**0.00**	-100
United States Virgin Islands	0	-1.5	-0.5	**-2**							H	0.25	**0**							**0.00**	-100
Uruguay	0	-1.5	-0.5	**-2**							H	0.25	**0**							**0.00**	-100

WHO rep: numbers show total cases reported 2007- 16, H=historic case reporting (prior to 2007). D=diagnosis score; C=contemporariness score; DQ=data quality score; TC=total number of cases (weighted); CS=case number score. HE L = health expenditure level: L=low, M=med, H=high. HS1/2 = health system score 1/2. Misdiagnosis likelihood scores: YW=yaws, CL=cutaneous leishmaniasis, ONC=onchocerciasis, LPR=leprosy, LF=lymphatic filariasis, TU=tropical ulcer, CMP=composite. ECS= evidence consensus score. *Score adjusted *post hoc*.

References for table S.5

1. Tai AYC, Athan E, Friedman ND, Hughes A, Walton A, O'Brien DP. Increased Severity and Spread of Mycobacterium ulcerans, Southeastern Australia. Emerging infectious diseases. 2018;24(1).
2. Sopoh GE, Barogui YT, Johnson RC, Dossou AD, Makoutode M, Anagonou SY, et al. Family relationship, water contact and occurrence of Buruli ulcer in Benin. PLoS neglected tropical diseases. 2010;4(7):e746.
3. Landier J, Boisier P, Fotso Piam F, Noumen-Djeunga B, Sime J, Wantong FG, et al. Adequate wound care and use of bed nets as protective factors against Buruli Ulcer: results from a case control study in Cameroon. PLoS neglected tropical diseases. 2011;5(11):e1392.
4. N'Krumah R TAS, Kone B, Cisse G, Tanner M, Utzinger J, Pluschke G, et al. Characteristics and epidemiological profile of Buruli ulcer in the district of Tiassale, south Cote d'Ivoire. Acta tropica. 2017;175:138-44.
5. Phanzu DM, Suykerbuyk P, Imposo DB, Lukanu PN, Minuku JB, Lehman LF, et al. Effect of a control project on clinical profiles and outcomes in buruli ulcer: a before/after study in Bas-Congo, Democratic Republic of Congo. PLoS neglected tropical diseases. 2011 a;5(12):e1402.
6. Bayonne Manou LS, Portaels F, Eddyani M, Book AU, Vandelannoote K, de Jong BC. [Mycobacterium ulcerans disease (Buruli ulcer) in Gabon: 2005-2011]. Medecine et sante tropicales. 2013;23(4):450-7.
7. Yeboah-Manu D, Aboagye SY, Asare P, Asante-Poku A, Ampah K, Danso E, et al. Laboratory confirmation of Buruli ulcer cases in Ghana, 2008-2016. PLoS neglected tropical diseases. 2018;12(6):e0006560.
8. Nakanaga K, Yotsu RR, Hoshino Y, Suzuki K, Makino M, Ishii N. Buruli ulcer and mycolactone-producing mycobacteria. Japanese journal of infectious diseases. 2013;66(2):83-8.
9. Kollie K, Amoako YA, Ake J, Mulbah T, Zaizay F, Abass M, et al. Buruli ulcer in Liberia, 2012. Emerging infectious diseases. 2014;20(3):494-6.
10. Ayelo GA, Anagonou E, Wadagni AC, Barogui YT, Dossou AD, Houezo JG, et al. Report of a series of 82 cases of Buruli ulcer from Nigeria treated in Benin, from 2006 to 2016. PLoS neglected tropical diseases. 2018;12(3):e0006358.
11. Beissner M, Arens N, Wiedemann F, Piten E, Kobara B, Bauer M, et al. Treatment Outcome of Patients with Buruli Ulcer Disease in Togo. PLoS neglected tropical diseases. 2015;9(10):e0004170.
12. South Sudan MoH. South Sudan Master Plan for Neglected Tropical Diseases. 2015.
13. Marion E, Obvala D, Babonneau J, Kempf M, Asiedu KB, Marsollier L. Buruli ulcer disease in Republic of the Congo. Emerging infectious diseases. 2014;20(6):1070-2.
14. Murphy HE. Buruli Ulcer In Sierra Leone: An Unique Undergraduate Elective Project Prize-winning Pathological Society Award for Best Elective Report 2011. J Pathol. 2013;229:S15-S.
15. Minime-Lingoupou F, Beyam N, Zandanga G, Manirakiza A, N'Domackrah A, Njuimo S, et al. Buruli ulcer, Central African Republic. Emerging infectious diseases. 2010;16(4):746-8.
16. Igo JD, Murthy DP. Mycobacterium ulcerans infections in Papua New Guinea: correlation of clinical, histological, and microbiologic features. The American journal of tropical medicine and hygiene. 1988;38(2):391-2.
17. World Health Organisation, editor 4th WHO Advisory Group Meeting on Buruli Ulcer2001.
18. Bradley DJea. Clinical Features and Treatment of Pre-Ulcerative Buruli Lesions: REPORT II OF THE UGANDA BURULI GROUP. Br Med J 1970.
19. Reynaud Y, Millet J, Couvin D, Rastogi N, Brown C, Couppie P, et al. Heterogeneity among Mycobacterium ulcerans from French Guiana revealed by multilocus variable number tandem repeat analysis (MLVA). PloS one. 2015;10(2):e0118597.
20. Walsh DS, Eyase F, Onyango D, Odindo A, Otieno W, Waitumbi JN, et al. Short report: Clinical and molecular evidence for a case of Buruli ulcer (Mycobacterium ulcerans infection) in Kenya. The American journal of tropical medicine and hygiene. 2009;81(6):1110-3.
21. dos Santos JL. Mycobacterium ulcerans infection in Brazil. MJA. 2007.
22. Guerra H, Palomino JC, Falconi E, Bravo F, Donaires N, Van Marck E, et al. Mycobacterium ulcerans disease, Peru. Emerging infectious diseases. 2008;14(3):373-7.

23. Kibadi K, Panda M, Tamfum JJ, Fraga AG, Longatto Filho A, Anyo G, et al. New foci of Buruli ulcer, Angola and Democratic Republic of Congo. Emerging infectious diseases. 2008;14(11):1790-2.
24. Al Ramahi JW, Annab H, Al Karmi M, Kirresh B, Wreikat M, Batarseh R, et al. Chronic cutaneous mycobacterial ulcers due to Mycobacterium ulcerans (Buruli ulcer): the first indigenous case report from Jordan and a literature review. International journal of infectious diseases : IJID : official publication of the International Society for Infectious Diseases. 2017;58:77-81.
25. Bessis D, Kempf M, Marsollier L. Mycobacterium ulcerans disease (Buruli ulcer) in Mali: A new potential African endemic country. Acta Derm Venereol. 2015;95(4):489-90.
26. Coloma JN, Navarrete-Franco G, Iribe P, Lopez-Cepeda LD. Ulcerative cutaneous mycobacteriosis due to Mycobacterium ulcerans: report of two Mexican cases. International journal of leprosy and other mycobacterial diseases : official organ of the International Leprosy Association. 2005;73(1):5-12.
27. Faber WR, Arias-Bouda LM, Zeegelaar JE, Kolk AH, Fonteyne PA, Toonstra J, et al. First reported case of Mycobacterium ulcerans infection in a patient from China. Transactions of the Royal Society of Tropical Medicine and Hygiene. 2000;94(3):277-9.
28. Komolafe OO. Buruli ulcer in Malawi - a first report. Malawi medical journal : the journal of Medical Association of Malawi. 2001;13(3):37-8.
29. Faber WR, de Jong B, de Vries HJ, Zeegelaar JE, Portaels F. Buruli ulcer in traveler from Suriname, South America, to the Netherlands. Emerging infectious diseases. 2015;21(3):497-9.
30. Ouoba K, Sano D, Traore A, Ouedraogo R, Sakande B, Sanou A. Cutaneous mycobacterium ulcerans ulcers in Burkina Faso: Six cases report with literature review. [French]. 1998.
31. Southern PM. Probable Buruli Ulcer Disease in Honduras. Open forum infectious diseases. 2016;3(2):ofv189.
32. Gordon D, Zelalem M, Schutze GE, Bilcha K. Suspected Buruli ulcer of the face: a case report from Ethiopia. The Pediatric infectious disease journal. 2014;33(3):323-5.
33. Stanford JL. Immunodiffusion Analysis of Strains of Mycobacterium-Ulcerans Isolated in Australia, Malaya, Mexico, Uganda and Zaire. Journal of Medical Microbiology. 1973;6(3):405-8.
34. Behrouznasab K, Razavi MR, Seirafi H, Nejadsattari T, Amini K, Amini K. Detection of mycobacterial skin infections by polymerase chain reaction (PCR) amplification of deoxyribonucleic acid (DNA) isolated from paraffin-embeded tissue. Afr J Microbiol Res. 2012;6(2):279-83.
